# Influence of MXene and TiO_2_ on the Performance of Microalgae-Derived Ru-Based Catalysts for CO_2_ Hydrogenation to Methane

**DOI:** 10.1021/acscatal.5c04285

**Published:** 2025-08-19

**Authors:** Agnieszka Sidorowicz, Thomas Wicht, Michael Stöger-Pollach, Roberta Licheri, Giacomo Cao, Alessandro Concas, Günther Rupprechter

**Affiliations:** † Interdepartmental Centre of Environmental Engineering and Sciences, 3111 University of Cagliari, Cagliari, 09123 Cagliari, Italy; ‡ Institute of Materials Chemistry, 27259 TU Wien, Getreidemarkt 9/BC, Vienna, 1060 Vienna, Austria; § University Service Center for Transmission Electron Microscopy, TU Wien, Stadionallee 2/057-02, Vienna, 1020 Vienna, Austria; ∥ Department of Mechanical, Chemical and Materials Engineering, University of Cagliari, Piazza d’Armi, Cagliari, 09123 Cagliari, Italy; ⊥ Center for Advanced Studies, Research and Development in Sardinia (CRS4), Loc. Piscina Manna, Building 1, Pula, 09050 Pula, Italy

**Keywords:** ruthenium, MXene, microalgae, CO_2_ hydrogenation, SMSI effect

## Abstract

Controlling the selectivity of CO_2_ hydrogenation to produce value-added fuels and chemicals is an actual challenge in catalysis research. The exact mechanisms underlying selectivity control often remain poorly understood, slowing the design of more efficient catalysts. In this study, we investigated RuO_2_ nanoparticles supported on MXene or TiO_2_ for CO_2_ hydrogenation at atmospheric pressure. Microalgal extracts were incorporated in the synthesis to explore their influence on catalyst properties, such as surface area, morphology, and elemental distribution. Although lower surface area and less uniform RuO_2_ dispersion were observed on MXenes than on TiO_2_, after reductive pretreatment Ru/MXene exhibited superior catalytic activity, demonstrating that its unique textural properties and active site availability compensated for the lower surface area. A reducibility study revealed that MXene-supported catalysts undergo a more complex reduction process than those with TiO_2_ as the support. Additionally, bridge adsorption sites on MXene likely contributed to the enhanced CO_2_ hydrogenation activity, whereas TiO_2_ seemed to present a twin CO binding environment. Higher Ru loading on MXene increased the methane selectivity and conversion, whereas lower loading favored CO formation, highlighting the importance of optimizing catalyst loading. *Operando* diffuse reflectance infrared Fourier transform spectroscopy analysis revealed the critical role of methoxy intermediates in affecting the catalytic pathway, suggesting the potential for tuning synthesis conditions to improve yields. A partial encapsulation of Ru on MXene enhances the catalytic performance, while the stronger SMSI effect on TiO_2_ leads to complete encapsulation, reducing the catalytic efficiency. The findings underscore the promise of MXene as a support material for metal catalysts in CO_2_ hydrogenation toward environmentally friendly fuel production.

## Graphical abstract

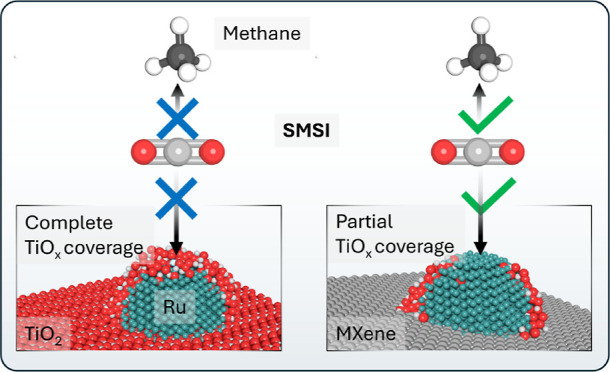

## 1 Introduction

The reaction of green hydrogen (H_2_), obtained via renewable-powered electrolysis, with carbon dioxide (CO_2_) captured directly from the atmosphere or flue gases, enables the production of so-called electro-fuels (e-fuels) that can help mitigate greenhouse gas emissions in several hard-to-electrify sectors like road and air transportation.^1^ Indeed, CO_2_ emissions from e-fuels’ combustion are equivalent to what was captured during their production, theoretically allowing the achievement of net-zero emission. According to recent estimates, the replacement of fossil fuels with e-fuels might lead to CO_2_ emission reduction, being up to 70% greater than traditional carbon capture and storage strategies.^2^ Moreover, when compared to pure H_2_, e-fuels represent a safer and energy-denser option for storing intermittent renewable electricity, enhancing energy system flexibility and enabling higher renewable energy exploitation.^3^ Among e-fuels, synthetic methane (CH_4_) is apparently most compatible with the existing natural gas infrastructure, allowing it to be distributed through established pipelines and storage systems in urban and rural areas. This compatibility lowers the need for new investments, enabling a faster and more cost-effective transition to sustainable energy carriers.

The reaction between H_2_ and CO_2_ is also well-recognized as a viable way to produce synthetic CH_4_ for the sustainment of future crewed missions to Mars.^4^ Indeed, H_2_ can be produced by electrolyzing Martian water, while CO_2_ is the main component (∼95 vol %) of the Martian atmosphere.^5^ Ruthenium (Ru) is a highly effective catalyst for it, which can be sourced from meteorites, offering a unique avenue for acquiring the precious metal.^6^ Subsequently, the synthesis methodology can not only reduce reliance on terrestrial mining but also align with future space exploration goals, where in situ resource utilization (ISRU) may play a crucial role.

To reduce expenses, nanoparticles (NPs) of Ru or RuO_2_ are typically placed on supports to maximize the surface to volume ratio, overall improving catalytic efficiency while maintaining the economical aspect of the synthesis.^7^ Alumina, which is widely used as a support, leads to weaker metal–support bonding than other supports,^8^ resulting in potential sintering of Ru particles at high temperatures, lowering activity. Silica, although chemically inert, often suffers from poor thermal stability and limited metal–support interaction, which can result in less efficient catalysis.^8,9^ Ru/TiO_2_ exhibits higher activity at lower temperatures than Ru/alumina^10^ and the excellent performance of this catalyst in CO_2_ or CO hydrogenation is attributed to specific metal–support interactions and interfacial sites.^10–13^ Various models have been suggested to clarify the crucial role played by TiO_2_ in CO_2_ methanation reactions, including a strong electronic effect of TiO_2_ on the Ru activity, which generally occurs after high-temperature pretreatment, facilitating C=O bond dissociation, and therefore increasing the overall activity.^14,15^

Recently, a versatile class of two-dimensional transition-metal carbides and nitrides (MXenes) has gained attention as a support in photocatalytic CO_2_ hydrogenation, offering exceptional electron transfer capabilities and strong metal–support bonding.^16,17^ MXenes have also been studied in a variety of other applications including thermal- and electro-catalysis.^18,19^ One of the biggest challenges in the synthesis concerns excessive water use to obtain MXene flakes. A recent study addressed the problem by utilizing 75% less water in the synthesis while using low-cost sodium bicarbonate.^20^ In addition, fluorine in wastewater was recovered through the precipitation of cryolite, a valuable compound used mainly in aluminum refining, which significantly improved the economic aspect by valorizing potentially harmful byproducts.^20^ Moreover, considering practical aspects, MXenes are susceptible to oxidation or degradation, which can result in their transformation into TiO_2_, particularly under oxidative or high-temperature conditions, altering its properties, affecting its catalytic performance.^21^ While there is extensive literature on the catalytic activity of Ru/TiO_2_,^22–24^ the effects of MXene support transformation into TiO_2_ have not been thoroughly studied. Existing studies focused on TiO_2_ phases—anatase, rutile, and their combination—but the interplay between the anatase phase and the original MXene structure in a combined material remains largely unexplored. Understanding this transformation and its impact on catalytic activity is important, as the MXene-TiO_2_ combination seems to exhibit unique properties not present in the TiO_2_ phases alone.

The innovative approach proposed herein involves upcycling waste from microalgae lipid extraction to prepare catalysts for CO_2_ hydrogenation. Recent advances in CO_2_ methanation have highlighted the effectiveness of Ru-based catalysts due to their high activity and selectivity at relatively low temperatures. Supports such as TiO_2_, Al_2_O_3_, and carbon-based materials have been widely studied to improve dispersion and stability.^25,26^ However, the use of 2D supports like MXenes, combined with sustainable synthesis methods involving bioderived agents from microalgae, remained unexplored. This study aims to address this lack by developing a Ru/MXene catalyst synthesized with microalgae-derived residues, thereby contributing to the development of more efficient and environmentally benign CO_2_ valorization pathways. The microalgal proteins, along with other biomolecules obtained from a typically discarded protein phase during Folch extraction, might act as capping agents controlling the growth of RuO_2_ nanoparticles and enhance their catalytic activity by preventing agglomeration, while promoting strong interactions between the nanoparticles and the support surface.^27,28^ Compared with conventional surfactants such as PVP, microalgae-derived residues offer several advantages: they are renewable, biodegradable, and rich in functional groups that can aid in nanoparticle stabilization. Moreover, their natural origin reduces the environmental footprint and simplifies postsynthesis treatment, as they can be more easily decomposed or removed without introducing synthetic residues that may interfere with catalytic performance.^29^ The catalytic hydrogenation of CO_2_ to methane is a promising carbon recycling and energy storage strategy. While Ru-based catalysts have been widely studied for this purpose, efforts to improve their sustainability and performance often rely on conventional supports and synthetic surfactants. Herein, we present a dual innovation: the use of microalgae-derived residues as a renewable templating and stabilizing agent and the integration of MXene as a support. Compared with traditional synthetic approaches, this route reduces environmental impact and enhances structural control. After anchoring nanoparticles to the support, MXene was further oxidized to obtain TiO_2_ anatase. Materials with different Ru loadings were characterized and tested for CO_2_ hydrogenation in contrast to commercial TiO_2_ anatase. Integrating microalgae-derived RuO_2_ with MXene and/or TiO_2_ supports not only tackles CO_2_ emissions but also bridges resource management, paving the way for sustainable advancements. While speculative, the use of bioderived residues and structurally robust supports highlights a sustainable synthesis approach that also aligns conceptually with ISRU strategies envisioned for long-term extraterrestrial missions such as CO_2_ utilization on Mars.^30^ Further studies are clearly needed to evaluate the feasibility and performance of such systems under specific conditions.

## 2 Materials and Methods

### 2.1 Catalyst Synthesis

MXene was prepared following methodology described previously.^20^ Briefly, 25 g of the Ti_2_AlC MAX phase (Nanografi Nano Technology Inc.) was mixed with 250 mL of 10% HF for 24 h at room temperature. Then, the sediment was isolated through centrifugation at 4000 rpm for 10 min and treated with 1000 mL of 0.05 M NaHCO_3_ to adjust the pH to neutral (7.0). Then, after centrifugation at 4000 rpm for 10 min, the sediment was similarly washed two times in 1000 mL of Milli-Q H_2_O in total. After washing, 500 mL of Milli-Q H_2_O was added, and the suspension was sonicated in a water bath for 1 h and centrifuged at 4000 rpm for 5 min. The product was then dried overnight at 70 °C in an oven. The reference anatase TiO_2_ NPs were supplied by Sigma-Aldrich and calcined in air at 600 °C for 3 h.

For the next steps of synthesis, a microalgae extract was prepared, as shown in Figure S1. *Chlorella vulgaris* (CCALA 902) culture was cultivated in Bold Basal Medium supplemented with 60 mM NaHCO_3_, at 25 °C with stirring (250 rpm) and 70 μmol m^–2^ s^–1^ photosynthetic photon flux density. After reaching the logarithmic growth phase (OD_750_ around 0.5–0.6) the biomass was separated from the medium through centrifugation at 2000 rpm for 5 min and dried at room temperature. Then, microalgal biomass was subjected to the Folch lipid extraction.^31^ First, 5 g of biomass powder was combined with 100 mL of methanol at 4 °C overnight followed by sonication in an ultrasonic bath for 30 min. After sonication, 200 mL of chloroform was added, and the solution was stirred for 1 h at 300 rpm. The biomass was separated through centrifugation at 4000 rpm for 10 min, and phase separation was induced by adding 60 mL of 0.88% KCl solution to the mixture. The mixture separates into two distinct phases: the lower chloroform (nonpolar) phase, which primarily contains neutral lipids, phospholipids, and other hydrophobic compounds, and the upper water (polar) phase, which retains hydrophilic molecules such as proteins, sugars, salts, and some polar metabolites from the microalgae. Using the polar phase for nanoparticle synthesis is important due to the presence of hydrophilic molecules, ionic species, and stabilizing agents that can influence nanoparticle formation, growth, and stability. In aqueous or polar environments, nanoparticles can be synthesized under controlled conditions where precursors dissolve efficiently, facilitating uniform nucleation and controlled growth while preventing aggregation to ensure that nanoparticles remain well-dispersed and stable. In a typical extraction methodology, the lower phase is usually processed to obtain lipids and the upper part is removed. In the current study, the upper part was collected, combined with 100 mL of Milli-Q H_2_O and subjected to evaporation using a rotary evaporator until methanol was removed. The resulting liquid was stored at 4 °C for further processing.

Finally, the impregnated catalysts were prepared following the methodology depicted in Figure S2. Initially, 10 mL of the polar phase extract (water-soluble compounds) was combined with 290 mL of Milli-Q H_2_O and heated to 50 °C while stirring at 250 rpm. Next, either 198 mg or 99 mg of RuCl_3_ (Sigma-Aldrich) was added to the extract, aiming at two different *nominal* Ru loadings (i.e., 3.1 and 5.9 wt %). The calculations of the nominal loadings are included in Supporting Information Note 1. After 15 min, an excess of 5% ammonia was used to increase the pH to 8, and the reaction continued for 3 h. Then, the colloid containing RuO_2_ nanoparticles was combined with 1.5 g of the support, and the flask was stirred for 1 h at 50 °C and left overnight at room temperature for maturation. The product was separated through centrifugation at 4000 rpm for 10 min and washed twice with 450 mL of Milli-Q H_2_O each cycle. At the end of the synthesis procedure, the product was dried overnight at 90 °C and calcined in air at 500 °C for 3 h to induce the structural changes of MXene and remove impurities, yielding a precatalyst of RuO_2_ on different supports.

### 2.2 Characterization

The actual chemical composition of the samples was determined by X-ray fluorescence (XRF) spectrometry using a PANalytical AxiosmAX WD-XRF system sequential spectrophotometer equipped with a rhodium tube as the source of radiation. XRF measurements were performed on pressed pellets with sample included in approximately 10 wt % of wax. The samples were irradiated with X-rays, causing the elements within the sample to emit secondary fluorescent X-rays, which were detected and analyzed to identify the specific elements present and their relative abundances. Quantitative analysis was performed using the fundamental parameter method, which corrects for matrix effects and provides accurate concentration values for each element. The data were processed to determine the weight percentages of the elements, enabling a detailed comparison of the elemental composition across different catalyst samples and supports.

X-ray photoelectron spectroscopy (XPS) was employed to analyze the surface elemental composition of the catalyst samples, specifically focusing on the oxidation states of the detected elements. XPS spectra were acquired at room temperature in a UHV chamber (base pressure < 3 × 10^–10^ mbar) equipped with a Specs XR50 high-intensity nonmonochromatic Al/Mg dual anode and a Phoibos 100 hemispherical electron energy analyzer with a multichannel plate detector. All measurements were performed using the Al anode at 1486.6 eV, at normal emission geometry, and in fixed analyzer transmission with a pass energy of 20 eV. The spectra were calibrated to the C 1s peak at 284.8 eV and analyzed using CasaXPS software. Quantitative analysis was performed to determine the atomic percentages of the elements on the surface.

X-ray diffraction (XRD) was utilized to characterize the crystalline phases, phase purity, and structural properties of the catalyst samples. The measurements were obtained by using an X-ray diffractometer (D8 Advance, Bruker AXS, and Philips XPERT-PRO). The samples were prepared by mounting the powdered catalysts onto a standard XRD sample holder. The X-ray beam was directed at the sample, and the diffracted X-rays were measured as a function of the angle 2θ. The scanning was performed with a diffraction angle between 20° and 80° at 40 kV and 30 mA using Cu Kα (λ = 1.54 Å) radiation. Data acquisition was performed with a step size of 0.25° and a scan speed of 4.5° per minute. The dwell time per step was set at 0.26 s to optimize the signal-to-noise ratio while maintaining reasonable throughput. The obtained diffraction patterns were analyzed using the Diffrac.Eva software v.6.1.0.4 and HighScore Plus (v5.1) software to identify the crystalline phases present by comparing the observed peaks with standard reference patterns from the Crystallography Open Database (COD) and Joint Committee on Powder Diffraction Standards (JCPDS) database. The crystalline size was calculated based on the Debye–Scherrer equation.

N_2_ adsorption–desorption isotherms were recorded by using an ASAP 2020 instrument (Micromeritics Inc., USA). Before each run, a known mass sample (ca. 0.18 g) was heated to 300 °C under vacuum for 2 h. After degassing, the samples were exposed to nitrogen (N_2_) gas at a liquid nitrogen temperature (−196 °C). The adsorption and desorption isotherms were recorded by measuring the volume of N_2_ adsorbed at different relative pressures (*P*/*P*_0_) ranging from 0.05 to 0.99. The Brunauer–Emmett–Teller (BET) surface area was calculated from the linear portion of the adsorption isotherm, typically in the relative pressure range of 0.05 to 0.3, using the BET equation. In addition to the surface area, the Barrett–Joyner–Halenda (BJH) method was employed to calculate the pore size distribution and total pore volume from the desorption branch of the isotherm.

The surface morphology was studied using scanning electron microscopy (SEM). The specimens were sputter-coated with an 8 nm layer of palladium and gold. The samples were mounted on carbon adhesive tabs and placed in a microscope chamber. The surface morphology was observed using a FEG-250, Quanta instrument with an accelerating voltage of 5 kV. In addition to qualitative analysis, energy-dispersive X-ray (EDX) Spectroscopy was employed in conjunction with SEM to obtain elemental mappings, allowing for the visualization of the spatial distribution of elements across the catalyst surface.

The detailed structural and morphological characteristics of the catalyst samples were investigated using transmission electron microscopy (TEM) performed by using a FEI TECNAI F20 field emission microscope equipped with a GATAN GIF Tridium energy filter and a GATAN Rio16 CMOS camera. Selected area electron diffraction (SAED) patterns were also recorded to confirm the crystallinity and to determine the crystal structure of the materials.

H_2_-TPR measurements were performed by using a Belcat instrument (MicrotracBEL Corp.) equipped with a thermal conductivity detector (TCD) for monitoring hydrogen consumption. As pretreatment, 50 mg of the catalyst was placed between quartz wool plugs and heated to 500 °C in 20 vol % O_2_ in Ar (heating rate 10 °C/min) to remove adsorbed impurities and moisture. After cooling, the sample was purged for 30 min with 100 vol % Ar. The H_2_-TPR measurements were carried out in a flow of 5 vol % H_2_ in Ar (total flow: 50 mL/min) up to 600 °C. The TPR profile was obtained by plotting the TCD signal against the temperature, allowing for the identification of reduction peaks corresponding to different reducible species within the catalyst. For comparative analysis, H_2_-TPR profiles were also obtained for the pure support materials without the active metal, allowing for identification of any support-related reduction events.

Hydrogen chemisorption measurements were performed using a BELCAT-B instrument (MicrotracBEL Corp.) to quantify surface-accessible Ru atoms and calculate the dispersion. Approximately 55 mg of catalyst was placed between quartz wool plugs in the BELCAT reactor system. The sample was pretreated in situ under a flow of 5% H_2_/Ar (50 mL/min) at 550 °C for 30 min to ensure complete reduction of the active metal species. After pretreatment, the catalyst was purged with argon (50 mL/min) at the same temperature for 10 min to remove residual gases and then cooled to 100 °C under flowing argon. Hydrogen chemisorption was carried out using the pulse injection method, where 74.7 μL H_2_ per pulse was injected sequentially into the carrier stream until no further uptake was detected, indicating surface saturation. After correcting for baseline and nondissociative adsorption, the total amount of chemisorbed hydrogen was determined from the cumulative uptake. A stoichiometric factor of H_2_/Ru = 1:2 was used to calculate the number of surface-exposed Ru atoms. The molar volume of gas at STP was used to convert the measured gas volume to moles, which was applied to calculate metal surface atoms per gram of catalyst, metal dispersion (%), surface metal atom density, and a known atomic surface density.

In situ diffuse reflectance infrared Fourier transform spectroscopy (DRIFTS) studies were carried out on a Bruker Vertex 70 spectrometer with a liquid N_2_-cooled MCT detector. A stainless-steel flow cell (Pike) features CaF_2_ windows and an oven. The inlet of the cell was connected to a gas manifold system with calibrated mass flow controllers to adjust the gas mixtures (pretreatment: 5 vol % H_2_ in Ar, total flow: 50 mL/min, adsorption: 5 vol % CO in Ar, total flow: 50 mL/min) and a mass spectrometer (Pfeiffer HiCube RGA, Pfeiffer Vacuum). Each sample was pretreated at 550 °C with a temperature ramp of 10 °C/min and kept at the maximum temperature for 30 min. For the CO adsorption step, 5 vol % CO in Ar was fed into the cell at a flow rate of 50 mL/min for 30 min up to adsorption saturation. For CO desorption, an Ar flow (50 mL/min) was purged into the cell to remove adsorbed CO. DRIFTS spectra were recorded with 4 cm^–1^ resolution using OPUS 6.5 software by averaging 64 scans to achieve a good signal-to-noise ratio.

### 2.3 Catalytic Testing

Before the catalytic measurement, a precatalyst (20 mg) was placed between quartz wool plugs and pretreated with 5% H_2_ at 550 °C for 30 min (heating rate 10 °C/min) to remove surface contaminants and activate the catalyst. Based on previous findings of O_2_-pretreatment lowering activity, the precatalyst was not oxidized before reduction.^22^ Then, the catalyst bed temperature was reduced to 250 °C as controlled by a thermocouple. For performing the catalytic reaction, a gas mixture of the following composition was introduced: 5% CO_2_, 20% H_2_ and 75% Ar, at 1 bar, with a total flow of 50 mL/min. The catalytic activity was tested at temperatures of 300 °C to 550 °C, and the effluent gases were continuously analyzed using a gas chromatography–mass spectrometry (GC + MS) system.

The GC (Agilent Technologies) was equipped with a capillary column suitable for separating light hydrocarbons and permanent gases as well with a TCD and flame ionization detector. GC data acquisition and product quantification were performed using Agilent Chemstation software (B.04.03), allowing for real-time monitoring of the catalytic performance under steady-state conditions. Simultaneously, a quadrupole mass spectrometer (QMS, Prisma Plus QMG 220, Pfeiffer Vacuum) was operated in electron ionization mode to detect reaction products online, including methane (CH_4_) and carbon monoxide (CO). The retention times and mass spectral data were used to accurately identify the compounds formed during the reaction. The conversion of CO_2_ and the selectivity toward different products were calculated based on the calibrated areas of the GC peaks corresponding to each product with equations provided in Supporting Information Note 2.

The ongoing reaction was studied by *operando* DRIFTS measurements, performed using the setup described above. The reaction chamber was fitted with CaF_2_ windows, allowing infrared light to pass through the sample while maintaining the desired gas flow and temperature conditions. Prior to the measurements, the precatalyst was loaded into the chamber and pretreated/reduced under a flow of 5% H_2_/Ar at 550 °C for 30 min (10 °C/min). After the mixture cooled to the desired reaction temperature, the gas flow was switched to a mixture of CO_2_ and H_2_ (1:4 ratio) at a total flow rate of 50 mL/min, simulating the conditions of CO_2_ hydrogenation. During the DRIFTS experiments, spectra were collected using OPUS 6.5 software with a resolution of 4 cm^–1^, accumulating 64 scans per spectrum. The background spectrum was recorded under pure Ar flow at the reaction temperature, and all spectra were normalized against this background to isolate the signals corresponding to the species adsorbed on the catalyst surface. The spectral region of interest (4000–1000 cm^–1^) allowed us to monitor the formation and evolution of surface intermediates throughout the reaction. The reactor effluent was continuously analyzed by GC + MS so that the *operando* DRIFTS data were directly correlated with the catalytic activity and selectivity under relevant reaction conditions.

## 3 Results and Discussion

### 3.1 Synthesis and Composition

For the synthesis, RuCl_3_·3H_2_O as the most common stable and economical Ru compound was chosen.^32^ In the past, Ru catalysts were prepared by impregnation with RuCl_3_ as the precursor and water as the solvent, leading to high Ru dispersion. In contrast, with other solvents the dispersion was lower and the catalytic activity decreased.^33^ In addition, chlorine from the precursor remaining after reduction can poison the catalyst, which can be mitigated by an appropriate washing method.^34^ The volume of water used for washing significantly influences the activity of Ru-based catalysts, with the use of, e.g., 900 mL water, resulting in the highest catalytic performance.^34^ The chlorine ions can also be eliminated by introducing NH_4_OH to the reaction mixture, with OH^–^ replacing the chloride in RuCl_3_, which enters the washing solution as Cl^–^. The side product, NH_4_Cl, formed in precipitation, is a valuable chemical and fertilizer, while catalytic activity is significantly increased and operation safety is improved. Therefore, the current synthesis conditions were optimized to obtain highly active catalysts, as shown in Figures S1 and S2.

Ammonium not only influences the chlorine ions but also increases the pH of the mixture. Increasing the pH of the solution promotes the formation/precipitation of Ru­(OH)_3_.^33^ When the concentration of ammonium is too high, the volume of the formed flocculent Ru­(OH)_3_ will quickly encapsulate Cl^–^, which makes the subsequent washing of Cl^–^ with deionized water difficult. During the synthesis process, the rate at which crystals form from ions in a specific orientation within the crystal lattice is referred to as the directional growth rate.^35^ The aggregate speed is determined mainly by the precipitation conditions and the supersaturation of the solute; the bigger the supersaturation, the quicker the aggregate speed. Ru­(OH)_3_ is unstable in the presence of air; however, in the presence of proteins from microalgae extract from the Folch extraction in the ammonium environment, it oxidizes to hydrous RuO_2_ deposited on the support by the impregnation method.^36^ When the aggregation rate exceeds the directional growth rate, particles precipitate in a largely amorphous form due to insufficient time to align directionally within the crystal lattice.

The actual bulk composition of the various catalysts was characterized by XRF analysis (Figure 1A,B; Table S1). The XRF spectra display the characteristic fingerprint region of Ru and confirm the successful deposition of Ru inside the pores, with the intensity of the Ru-specific peaks directly representing Ru loadings, indicating 3.3 and 5.2 wt % (metallic) Ru on MXene and 3.4 and 6.5 wt % (metallic) Ru on TiO_2_. Differences between nominal (aimed for) and actual (measured) loadings can occur due to Ru loss during synthesis and washing steps and varying interaction strengths between RuO_2_ and supports. Clearly, after calcination, Ru is present as RuO_2_ in the precatalyst, but this does only slightly change the loading values (Supporting Information Note 3, Table S2). Therefore, in the figures about calcined samples, the RuO_2_ loading is given. Before catalysis, RuO_2_ is in situ reduced to Ru (so Ru loadings are given). Describing the precatalyst (supported RuO_2_) before reduction/reaction is important as it provides a comprehensive understanding of the catalyst across multiple stages of preparation, enabling a comparison between its oxidized and reduced states. It also offers valuable insights into the uniformity of the nanoparticles in their oxidized state, which is crucial for attributing differences in catalytic performance to the reduction process or the support interactions rather than variations in initial nanoparticle characteristics.

**Figure 1:**
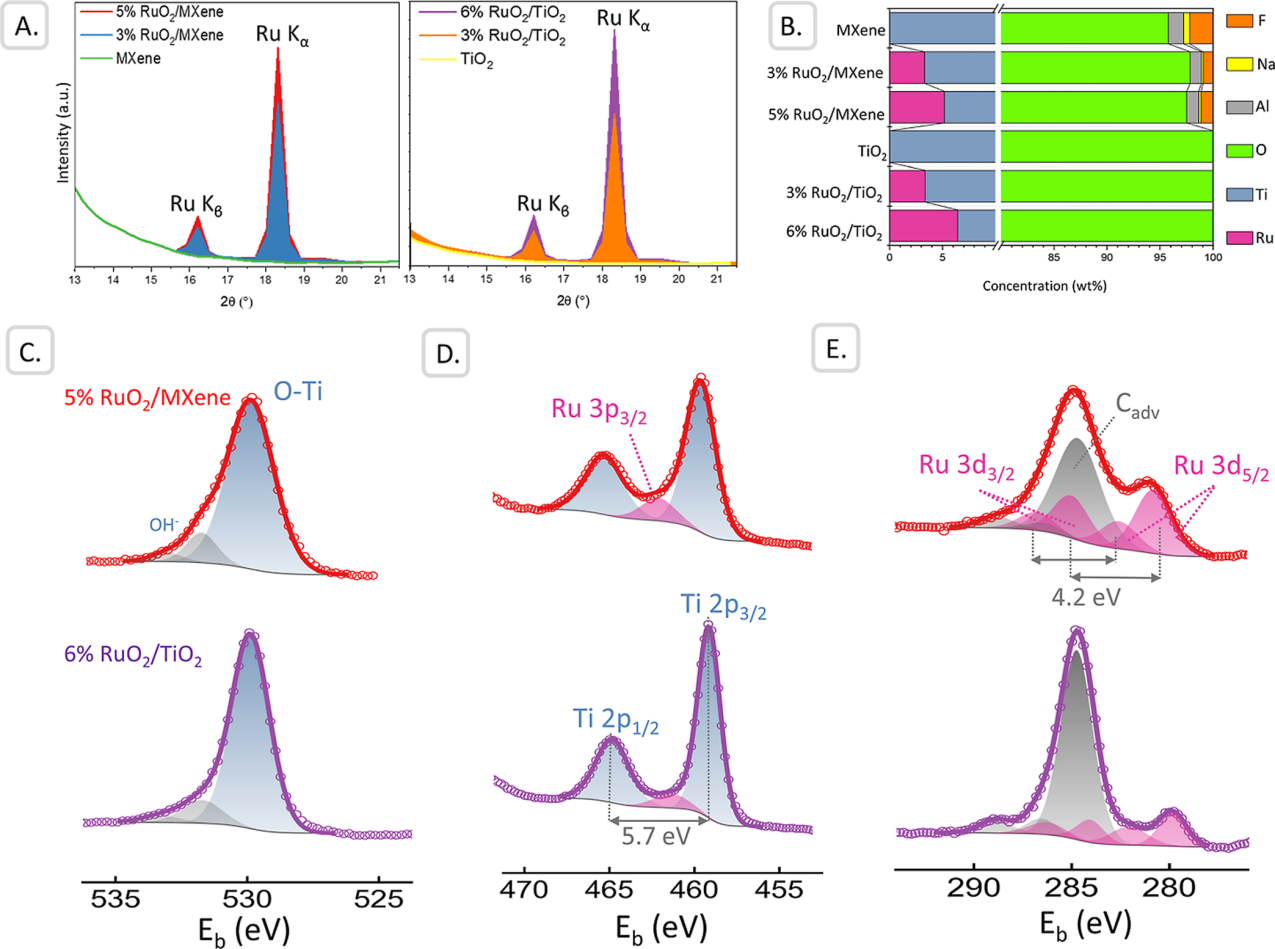
Elemental characterization of Ru-based catalysts: (A) fingerprint XRF Ru regions, (B) quantitative relative XRF composition, (C) XPS O 1s region, (D) XPS Ti 2p and Ru 3p region, (E) XPS C 1s and Ru 3d regions.

The higher content of sodium and fluorine observed for the MXene support without Ru can be attributed to the characteristics of the MXene synthesis process, as it was synthesized by MAX phase etching, leaving behind residual sodium and fluorine from the etchant solution (Table S1).^20^ When the active Ru component is loaded onto the MXene, the residual elements may be partially removed or diluted due to the washing and preparation steps involved in catalyst loading, resulting in lower levels of sodium and fluorine.

XPS analysis then characterized the surface composition of representative 5% RuO_2_/MXene and 6% RuO_2_/TiO_2_ (Figure 1C–E, Table S3). The O 1s region shows a pronounced peak at 530.0 eV for both samples (Figure 1C), as expected for O in TiO_2_ with a possible contribution of oxygen from RuO_*x*_.^37^ Additionally, shoulders at 531.5 eV indicate the O from the Ti–OH bond while the peak at approximately 533 eV can be attributed to water or organic residues. Considering the Ti 2p region (Figure 1D), for MXene samples, a main peak with binding energy of 459.0 eV was observed, typical of Ti^4+^ in TiO_2_ with doublet separation of 5.7 eV. The TiO_2_ support shows a similar pattern with a Ti 2p_3/2_ binding energy of 458.6 eV and doublet separation of 5.7 eV. Neither TiC at 455 eV nor any suboxides (<457.5 eV) were detected on the MXene surface, suggesting that it is likely present in subsurface layers. Instead, due to the calcination step, the surface seems to be fully oxidized, with the slight increase in binding energy indicating partial fluorination TiO_2–*x*_F_*x*_.^38^ Moreover, between the Ti 2p doublet, a small peak of Ru 3p_3/2_ arose at 461 eV, though additionally the intensity of Al_Kβ_ of the O 1s may contribute. Lastly, the C 1s and Ru 3d regions were deconvoluted (Figure 1E). Apart from adventitious carbon calibrated to 284.8 eV, additional carbon species at 286 and 289 eV indicated O-containing organic residues. Additionally, there were two more species at ≤282 eV, too low for any carbon species, especially as TiC was not detected in the Ti 2p region. Instead, the peaks can be assigned to Ru 3d_5/2_ and were fitted together with Ru 3d_3/2_ with a doublet separation of 4.2 eV.^39^ The need for at least two Ru 3d_5/2_ species indicates a variety of oxidation states, including metallic Ru^0^ at 279.8 eV for the 6% RuO_2_/TiO_2_ sample, with the reduction likely caused by X-ray exposure in vacuum. The spectra of F 1s, Cl 2p, and Al 2p were recorded and are presented in the Supporting Information (Figure S3). The F 1s spectrum shows two distinct features: at 685.0 eV matching with F in TiO_2–*x*_F_*x*_ with a shoulder at 686.4 eV of the Al–F bond, while 4.2–4.3 at. % of fluorine can be detected on the surface.^38^ The origin of the peak in the F 1s region of RuO_2_/TiO_2_ remains unclear. Fluorine is not listed as a trace element in the commercial TiO_2_ used and it was not detected by XRF analysis in the 6% RuO_2_/TiO_2_ sample. In addition, the peak position is shifted by −1 eV compared to that of the TiO_2–*x*_F_*x*_ component in 5% RuO_2_/MXene. As the area corresponds to no more than 0.5% of the sample (compared to >4% F in MXene), it was not taken into account for quantification. Importantly, no significant Cl 2p signal was detected, indicating the effective removal of chloride residues from the RuCl_3_ precursor during catalyst preparation and washing. The Al 2p spectrum exhibits signals near 120 eV, which can be attributed to contributions from the Al_2_O_3_ and Al–F species. This suggests that a portion of the aluminum from the original MAX phase remains in oxidized or fluoride-bound forms, despite the etching and delamination processes.

The surface atomic percentage of ruthenium in the catalyst system varies significantly due to the differing textural properties of the supports used. MXene, with its less extensive pore structure, facilitates a higher surface coverage of ruthenium atoms, leading to a greater atomic percentage. In contrast, TiO_2_, often characterized by its deeper and more abundant pores, allows for higher Ru dispersion within its porous network, which results in a lower surface concentration of ruthenium atoms, explaining the lower atomic percentage. The higher pore depth of TiO_2_ effectively increases the total surface area available for dispersion but lowers the surface density of ruthenium compared to more accessible surface sites on MXene.

### 3.2 Crystallographic Characterization

The phase composition and crystallite size of the calcined catalysts were studied by XRD (Figure 2A). The results confirmed the successful incorporation of RuO_2_ NPs into the support at different loadings and assessed the interaction between the support and active metal. The two peaks at 28.25° and 35.34° were attributed to the orthorhombic *Pnnm* (58) RuO_2_ structure (JCPDS 88-0323), specifically (110) and (011), respectively. The same pattern was previously obtained using *Acalypha indica* leaf extract, with alkaloids hypothesized to be crucial for the synthesis process.^40^ Interestingly, the crystallite size of the RuO_2_ NPs decreased with increasing loading, for both MXene and TiO_2_ supports. However, we acknowledge that this method provides only an approximate estimation and may be influenced by additional factors such as strain or instrument-related broadening. Future studies will aim at validating these findings and at providing more detailed insights into particle size and dispersion. As more active material was loaded, it might form smaller and more numerous crystallites to maximize its contact with the support surface, thereby enhancing the overall catalytic surface area. XRD spectra of the (pure) support revealed the presence of tetragonal TiO_2_ *I*4_1_/*amd* (141) anatase (JCPDS 79-1764) with major peaks at 25.52° and 48.25°, corresponding to (101) and (200), respectively. Additionally, MXene displayed TiC (cubic, *Fm*3̅*m* (225), COD 5910091) and Na_2_C_2_ (tetragonal, *I*4_1_/*acd*:2 (142), and COD 1521140) patterns. Although MXene and TiO_2_ supports shared the same TiO_2_ pattern, MXene retained its unique layered structure that includes a TiC component, which might contribute to its distinct characteristics and influence on catalytic behavior.

**Figure 2:**
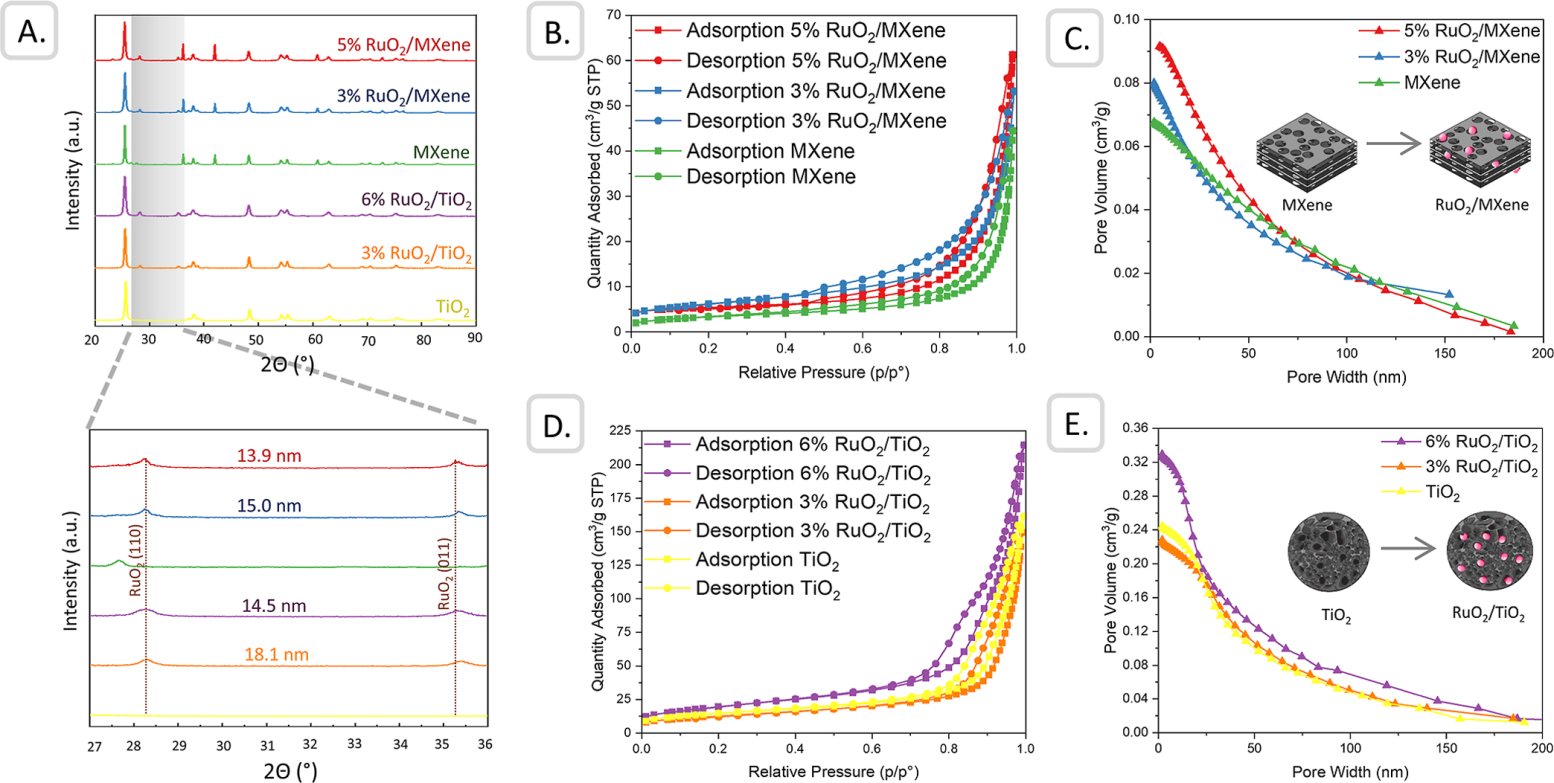
Structural and elemental characterization of RuO_2_/MXene and RuO_2_/TiO_2_ catalysts in the calcined state: (A) XRD pattern, (B–E) BET nitrogen adsorption isotherms with specific surface area and pore distribution of the catalysts.

A detailed structural characterization of the loaded and unloaded support is crucial for catalysts prepared by impregnation, especially when the support is introduced after the nanoparticles were formed.^33^ The process utilizes the support with a given shape and size, omitting the commonly studied step of catalyst shaping, allowing the choice of a suitable support that can provide the necessary physical structure of the catalyst, such as surface (11.5 m^2^/g for MXene, 50.4 m^2^/g for TiO_2_), pore (0.068 cm^3^/g for MXene, 0.245 cm^3^/g for TiO_2_), and thermal conductivity.^41^ RuO_2_ NPs are primarily distributed on the surface and within the pores of the support, offering high utilization efficiency, minimal usage, and reduced cost, which is particularly crucial for precious metal catalysts.^41^ Therefore, impregnation is a simple and economical method in comparison with other techniques such as chemical vapor deposition or sol–gel infiltration, widely used to prepare supported catalysts, especially low-content-supported precious metal catalysts. Although the impregnation method is straightforward, it can lead to issues such as uneven distribution of the active component, with a higher concentration on the outer surface and a lower concentration on the inner surface after drying.

### 3.3 Surface Area and Pore Structure Analysis

The Brunauer–Emmett–Teller (BET) analysis was employed to evaluate the specific surface area, pore volume, and pore size distribution of the synthesized calcined catalysts (Figure 2B–E). All samples exhibited the IUPAC type III isotherm, indicating mesopores in the as-prepared materials. The MXene catalyst without Ru loading exhibited a low BET surface area of 11.5 m^2^/g, with a total pore volume of 0.068 cm^3^/g (Table 1). The reduced surface area and increased pore size of the MXene support are indicative of its layered structure, which, while providing ample sites for metal loading, may result in a less accessible surface area than TiO_2_. The surface area increased to 21.5 m^2^/g upon 3 wt % Ru loading and to 18.2 m^2^/g upon 5 wt % Ru loading, accompanied by increased pore volume. The introduction of RuO_2_ via impregnation has the potential to increase the surface area; however, based on the calculations (Supporting Information Note 4), the increase is small (4.8 m^2^/g, 4.0 m^2^/g and 2.4 m^2^/g for 6, 5 and 3%, respectively). The observed trend may indicate that the RuO_2_ particle deposition process prevents the blockage of existing pores and generates new pore structures, possibly due to the interaction between RuO_2_ NPs and the support. As the loading of the active material increases, it can lead to the formation of additional pores or the widening of existing pores on TiO_2_ due to the physical displacement of the support material and the creation of void spaces between the dispersed active particles, preventing the collapse of pores and maintaining or even enhancing the structural integrity of the porous network.^42,43^ Furthermore, high loading levels can reorganize the support material, improving its porosity by creating interparticle voids and increasing the overall pore volume.

**Table 1:** BET Surface Area and Pore Structures of the Prepared Catalysts

catalyst	BET surface area (m^2^/g)	BJH adsorption cumulative pores volume (cm^3^/g)	BJH average pore width (nm)
MXene	11.5	0.068	27.6
3% RuO_2_/MXene	21.5	0.080	18.0
5% RuO_2_/MXene	18.2	0.092	29.1
TiO_2_	50.4	0.245	23.7
3% RuO_2_/TiO_2_	44.2	0.228	23.0
6% RuO_2_/TiO_2_	69.0	0.330	19.7

For TiO_2_, the BET surface area was approximately 50.4 m^2^/g, with a total pore volume of 0.245 cm^3^/g. These values are consistent with the mesoporous nature of TiO_2_, which is known to provide a high surface area to disperse active metal nanoparticles like RuO_2_.^44^ A similar increasing surface area/pore volume trend with increasing loading was observed as for MXene, suggesting effective dispersion of RuO_2_ NPs, leading to enhanced porosity and a greater number of active sites. However, a larger pore size of MXene could facilitate the diffusion of reactants and products during catalysis, potentially compensating for the lower surface area.^45^ The results suggest that while TiO_2_ represents a more conventional high-surface-area support, the distinct structural features of MXenes may provide advantages in catalytic applications, particularly with factors beyond the surface area.

### 3.4 Structural Analysis of Supported RuO_2_ NPs

Differences in morphology and RuO_2_ distribution between MXene and TiO_2_ supports of precatalysts were further observed by SEM and EDX mappings (Figure 3A,B,E,F), indicating several factors apart from the previously discussed specific surface area. The MXene-supported catalysts exhibited a typical accordion-like morphology, indicative of the layered structure of MXene.^46^ The SEM images of the TiO_2_-supported catalysts revealed a characteristic spherical particle shape of TiO_2_ with a relatively uniform size distribution. On MXene, the RuO_2_ nanoparticles exhibited a size distribution ranging from 10 to 40 nm, average 18.7 nm, while on TiO_2_ mild aggregation was occasionally observed with size range 13–28 nm, with the same average. The particle size distribution histograms of both samples are shown in Figure S4. The EDX maps highlighted the presence of Ti and O across both supports, confirming the integrity of the TiO_2_ phase. For both, carbon signals were detected, which might originate from the microalgal extract used during the synthesis or the carbon tape used for sample mounting. However, in the case of the pure MXene support, the mapping also revealed the presence of residual C, consistent with the TiC layers that were not completely oxidized during synthesis. This suggests that the MXene support maintains its unique layered structure, which may contribute to the better RuO_2_ dispersion than on TiO_2_, where RuO_2_ is located within the pores. In addition, the MXene support exhibited the presence of Al, in agreement with XRF, likely originating from the MAX precursor material used for synthesis.

**Figure 3:**
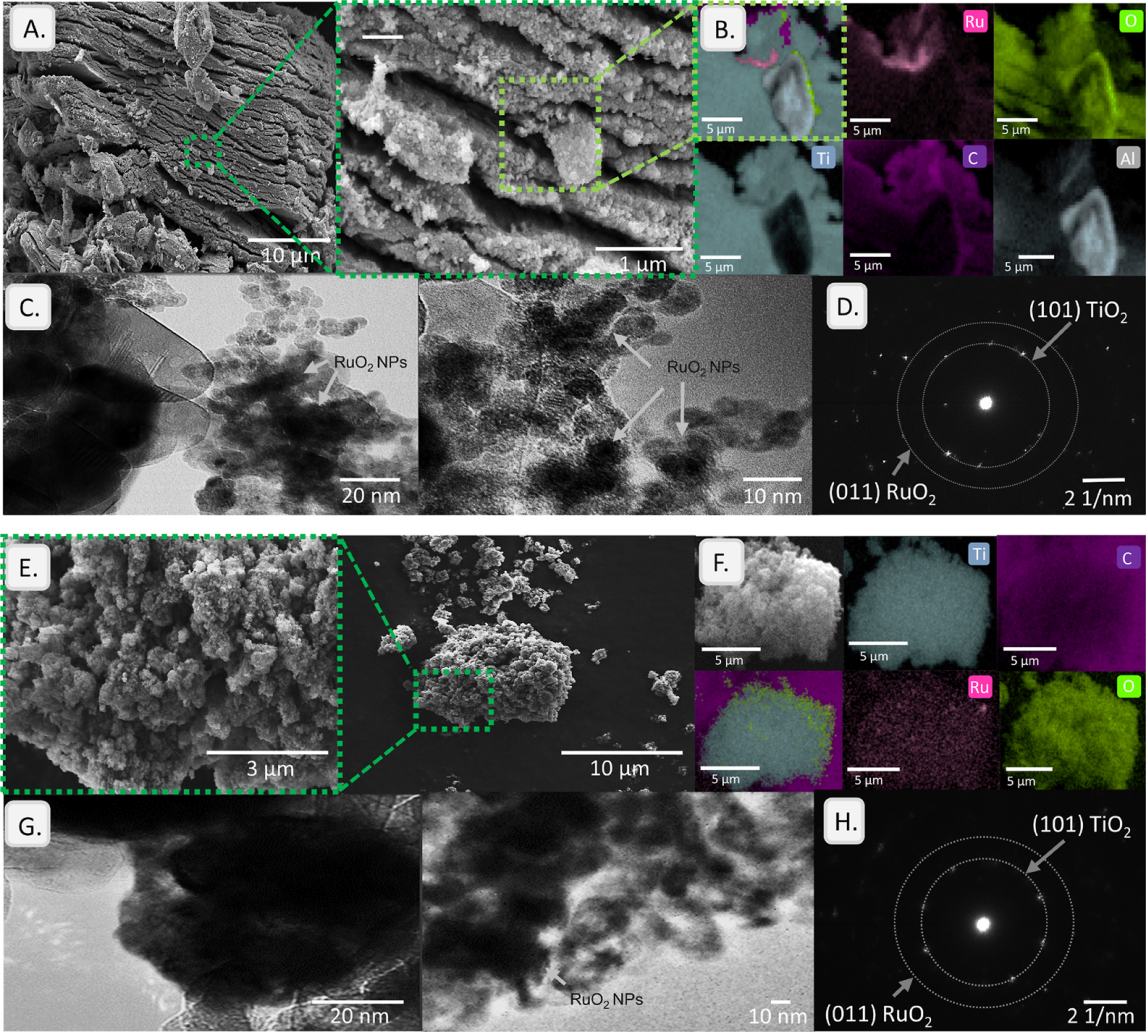
Surface morphology of the catalysts: (A) SEM images and (B) EDX mappings of 5% RuO_2_/MXene, revealing the spatial distribution of elements across the catalyst surface, with different colors representing the intensity of elemental signals. (C) TEM images and (D) SAED pattern of 5% RuO_2_/MXene. (E) SEM images and (F) EDX mappings of 6% RuO_2_/TiO_2_. (G) TEM images and (H) SAED pattern of 3% RuO_2_/TiO_2_.

TEM analysis provided more detailed insights into the size and dispersion of RuO_2_ NPs on the supports of the precatalysts (Figure 3C,G). The MXene-supported catalyst exhibited a distribution of RuO_2_ NPs on the support surface, with some particles appearing larger due to partial agglomeration. The surface localization indicates that the RuO_2_ nanoparticles are more accessible to the reactants, which should favor catalytic activity. On the TiO_2_-supported catalyst, RuO_2_ NPs were well-dispersed in the pores, potentially reducing the accessibility of these active sites, which may lower the overall activity. The difference in particle size distribution can be attributed to the textural properties of the supports, as the lower specific surface area of MXene may not effectively prevent nanoparticle coalescence during synthesis. The crystallinity of the RuO_2_ NPs and the supports was assessed by SAED (Figure 3D,H). In the case of the MXene-supported catalyst, the SAED pattern showed diffuse rings indicative of the less ordered nature of the MXene layers. However, distinct spots corresponding to the anatase phase of TiO_2_ and the RuO_2_ phase were still observed, indicating that during partial agglomeration, the RuO_2_ NPs maintained their phase purity. In the case of TiO_2_-supported catalysts, the SAED pattern exhibited sharp diffraction spots similarly corresponding to the anatase phase of TiO_2_ and the RuO_2_ phase, confirming the high crystallinity of both the support and the RuO_2_ NPs. The comparison of the SAED patterns suggests that the MXene support, while less crystalline than TiO_2_, still provides a suitable environment to anchor and maintain crystalline RuO_2_ NPs. The lower crystallinity of the MXene support may affect the interactions with the RuO_2_ NPs, potentially leading to catalytic behavior different from that of the TiO_2_-supported catalysts.

### 3.5 Reduction Behavior and Active Site Formation

The H_2_-TPR profiles of the RuO_2_ nanoparticles supported on MXene and TiO_2_ were recorded to evaluate the RuO_2_ reducibility (as the studied reaction includes H_2_) and to understand the interaction between RuO_2_ NPs and the supports (Figure 4A). During reduction, RuO_2_ with an orthorhombic *Pnnm* (58) crystal structure undergoes breaking of Ru–O bonds and rearrangement of Ru atoms into a more stable hexagonal close-packed (hcp) *P*6_3_/*mmc* structure characterized by a higher electronic conductivity. The MXene-supported catalysts displayed a more complex reduction profile with three distinct peaks. The first peak, observed at around 180 °C, is likely attributed to the reduction of RuO_*x*_ particles weakly interacting with the MXene surface.^47^ The second, broader peak, appearing at approximately 250 °C, indicates the reduction of RuO_2_ species more strongly interacting with the MXene layers.^47^ The third peak, seen at about 315 °C, is likely associated with the reduction of RuO_2_ species embedded within the MXene structure or those in closer contact with the Ti sites of the MXene support, which may require higher temperatures for reduction. As the Ru loading increased, the area of the reduction peaks increased, reflecting a higher quantity of reducible Ru species. However, the peak temperatures remained relatively unchanged, suggesting that the increase in loading did not significantly alter the nature of the metal–support interaction. Notably, an additional broader high-temperature peak around 550 °C was observed for the MXene-supported catalyst, even in the absence of RuO_2_ loading. The peak is likely related to the reduction of oxygen species inherent to the MXene structure, such as Ti–O, which requires a higher energy for reduction.

**Figure 4:**
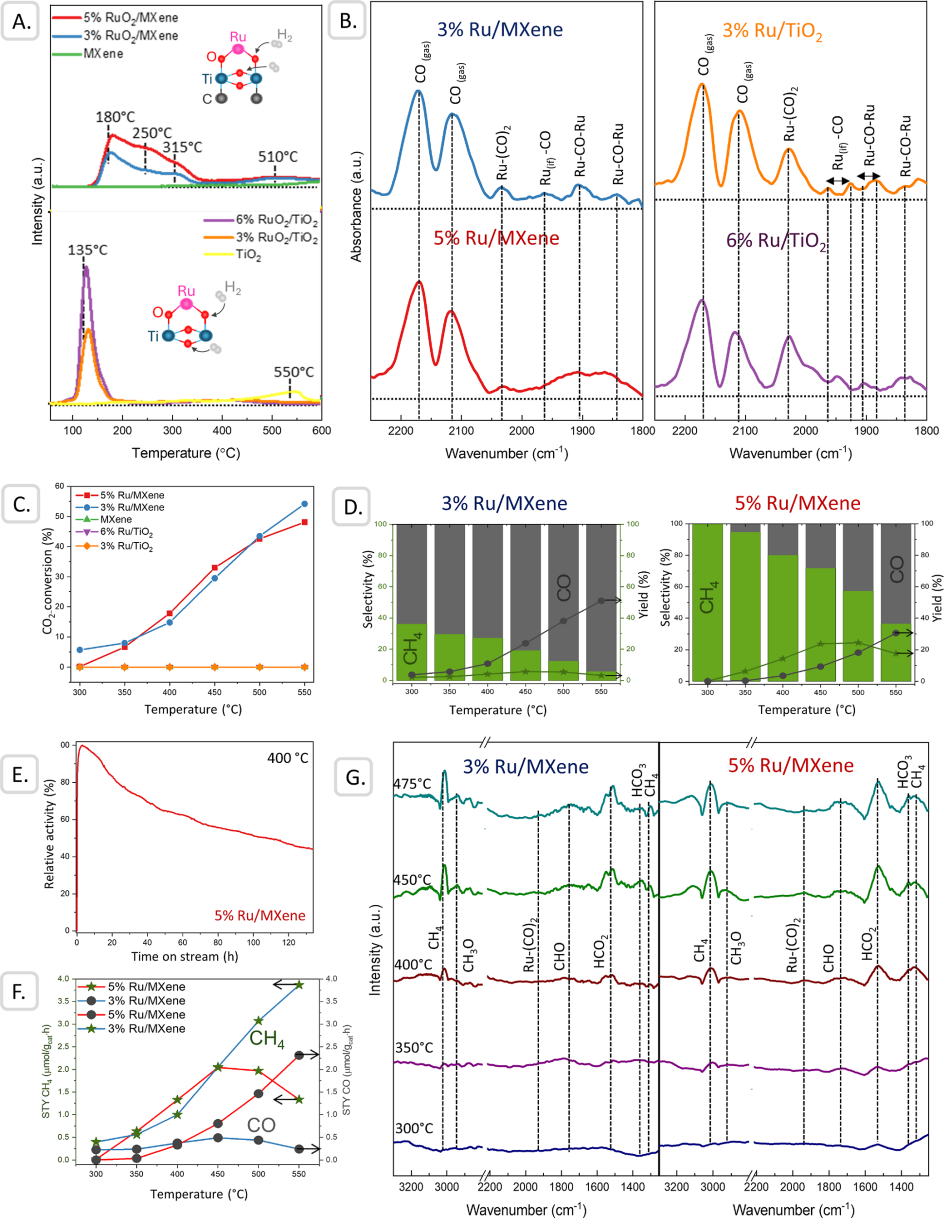
Active species and catalytic activity: (A) H_2_-TPR, (B) CO–DRIFTS, (C) CO_2_ conversion as a function of temperature of all tested samples, (D) selectivity and yield of CH_4_ and CO as a function of temperature of 3% Ru/MXene and 5% Ru/MXene, (E) relative activity of 5% Ru/MXene based on MS illustrating the stability of the product signal during CO_2_ hydrogenation, (F) space-time yield (STY) of CH_4_ and CO for 3% Ru/MXene and 5% Ru/MXene at different temperatures, (G) *operando* DRIFTS of 3% Ru/MXene and 5% Ru/MXene in a temperature range of 300–475 °C.

The TiO_2_-supported catalysts exhibited a single sharp reduction peak centered at around 135 °C, corresponding to the reduction of RuO_*x*_ to metallic Ru. The relatively low temperature of this peak suggests that RuO_2_ is weakly interacting with the TiO_2_ support, making it easily reducible. The broad peak at 510 °C in the H_2_-TPR profile of the TiO_2_ support likely corresponds to the reduction of more strongly bound oxygen species associated with the TiO_2_ surface. The disappearance of the 550 °C peak in the RuO_2_-loaded TiO_2_, compared to the unloaded TiO_2_, suggests a hydrogen spillover effect, where hydrogen atoms migrate from the metal catalyst to the TiO_2_ support, facilitating its reduction at lower temperatures.^48^ The spillover reduces the need for high-temperature reduction, resulting in peak absence. The TPR results highlight more diverse metal–support interactions in the MXene-supported catalysts, as indicated by the presence of multiple reduction peaks at higher temperatures than the TiO_2_-supported catalysts. The MXene support may stabilize the RuO_2_ species more effectively, leading to different catalytic properties in CO_2_ hydrogenation, where the reducibility critically influences the activity and selectivity.

A pretreatment (reduction) temperature at 200–800 °C was previously applied to optimize the catalytic activity of Ru on TiO_2_.^49^ The results showed that the extent of encapsulation of the Ru particles by TiO_*x*_ layers and the number of hydroxyl groups on the TiO_2_ surface increased upon increasing pretreatment temperature from 300 to 600 °C.^12,49^ However, due to the possible rearrangement of anatase to rutile at 600 °C, a reduction temperature of 550 °C was selected herein to maintain the same TiO_2_ phase in both tested supports for better comparison of activity.

### 3.6 Binding Chemistry and Active Site Distribution

To provide information about the nature and strength of metal–molecule interactions and the types of active sites present in the reduced catalyst after H_2_ pretreatment, CO was employed as a probe for CO–DRIFTS (Figure 4B). The CO–DRIFTS results revealed differences in the coordination environment of Ru on the MXene and TiO_2_ supports. The strong adsorption peaks centered at 2173 cm^–1^ and 2110 cm^–1^ were attributed to the R and P branches of the rotation vibrational spectra of gas-phase CO species.^50^ The broader and more intense peaks of CO binding with two Ru molecules (Ru–CO–Ru) at 1905 cm^–1^ and 1843 cm^–1^ on MXene suggest a higher degree of structural diversity in the Ru sites,^22^ likely due to the heterogeneous surface chemistry and multiple binding sites of the MXene. The presence of significant bridging CO adsorption at 1905 cm^–1^ and 1843 cm^–1^ on MXene suggests the variety of active sites for CO_2_ hydrogenation, potentially enhancing the ability to activate CO_2_ and H_2_ molecules. In contrast, the more intense peak at 2030 cm^–1^ of two CO molecules binding to Ru NPs (Ru–(CO)_2_)^22^ suggests a more uniform but potentially less versatile catalytic environment. A sharp, intense CO peak indicated that most of the Ru sites on TiO_2_ were available for CO adsorption, likely due to the dispersion of small Ru particles on the TiO_2_ surface. The intensity of single CO molecule adsorption at the interface between Ru and TiO_2_ (Ru_if_–CO) at 1926–1965 cm^–1^ was similar to that of MXene-supported catalysts.^22^ The differences in the CO adsorption behavior have important implications on the catalytic activity in CO_2_ hydrogenation (see below). Multiple CO binding modes on MXene may enhance the catalytic activity by facilitating the activation and conversion of CO_2_ into desired products by promoting more efficient electron transfer than the more uniform single CO binding on TiO_2_. Similar results were obtained before, with samples showing dominant one CO binding exhibiting the lowest activity, indicating that size and surface area do not play critical roles in enhancing the catalytic performance, whereas different surface atomic bonding configurations of Ru NPs directly modified their catalytic activity.^22^

### 3.7 Catalytic Performance in CO_2_ Hydrogenation: Insights from *Operando* DRIFTS

The catalytic performance in CO_2_ hydrogenation was evaluated for Ru-based catalysts supported on MXene or TiO_2_ after H_2_ pretreatment (Figure 4C). The results demonstrated significant differences in CO_2_ conversion, CH_4_ and CO selectivity, and thus CH_4_ and CO yield, and STY among the different catalysts. While the TiO_2_-supported catalysts were inactive, the MXene-supported Ru catalyst exhibited catalytic CO_2_ hydrogenation activity in the 300–550 °C range, with similar CO_2_ conversion of the two loadings increasing with temperature, reaching around 48–54% (Figure 4C). To evaluate the possible contribution of residual elements (such as Al, F, and Na) originating from the MXene synthesis to the observed catalytic activity, a control experiment was conducted using bare MXene under identical CO_2_ hydrogenation conditions, which only showed negligible CO_2_ conversion (<0.5%). The enhanced activity observed for Ru/MXene can thus be mainly attributed to the presence of Ru species and their interaction with the MXene support. A promotional effect of residual elements on Ru has been reported, but the Na loading for promoting CO_2_ methanation was ∼15-times higher than in our catalysts.^51^ The two active catalysts showed distinctly different selectivity toward CH_4_ and CO (Figure 4D, Table 2). The 5% Ru/MXene achieved a higher CH_4_ selectivity of 80% at 400 °C, which mimics the composition of natural gas.^52^ The highest observed CO selectivity was 63% at 550 °C. The 3% Ru/MXene catalyst showed a maximum CH_4_ selectivity of around 30%, with CO selectivity reaching around 94% at 550 °C. The higher Ru content might improve the adsorption and activation of reactants, driving the reaction pathway more efficiently toward methane formation, as observed before.^14^ The yield of CH_4_, defined as the amount of CH_4_ produced per mole of CO_2_ converted, was calculated based on the selectivity and CO_2_ conversion. The 5% Ru/MXene catalyst exhibited the highest CH_4_ yield of 24.4% at 500 °C, compared to 5.7% at 450 °C for the 3% Ru/MXene catalyst, while the highest CO yield was recorded at 30.5 and 51.0%, both at 550 °C, for 5% Ru/MXene and 3% Ru/MXene, respectively. The long-term stability of the 5% Ru/MXene catalyst was evaluated under continuous CO_2_ hydrogenation conditions for 134 h at 400 °C (Figure 4E). Product concentrations were normalized to the argon internal standard to correct for fluctuations in the flow rate and detector response. Normalized product signals were calculated as the ratio of the product peak intensity to the argon peak intensity. The catalyst exhibited maximum activity, followed by a gradual decline in activity, reaching 44% of the maximum activity at the end of the test. An exponential decay model was fitted to the activity profile to analyze the deactivation behavior (Supporting Information Note 5, Figure S5). The yielded parameters indicate that the catalyst retains approximately 39% of its initial activity, even after long operation. The catalyst is expected to reach within 1% of its residual activity (∼39.05%) after approximately 275 h. The plateau suggests that a fraction of active Ru sites remains stable over time, contributing to the overall performance.

**Table 2:** Overview of CO_2_ Conversion, CH_4_ and CO Selectivity, Yield, and STY for Different Ru Loadings on MXene Supports, at Different Temperatures

catalyst	CO_2_ conversion (%)	CH_4_ selectivity (%)	CO selectivity (%)	CH_4_ yield (%)	CO yield (%)	CH_4_ STY (μmol/g_cat_ × h)	CO STY (μmol/g_cat_ × h)
MXene	0.0	0.0	0.0	0.0	0.0	0.000	0.000
3% Ru/MXene-300 °C	5.7	36.2	63.8	2.1	3.6	0.224	0.396
3% Ru/MXene-350 °C	8.0	29.7	70.3	2.4	5.6	0.237	0.561
3% Ru/MXene-400 °C	14.8	27.3	72.7	4.0	10.8	0.375	0.997
3% Ru/MXene-450 °C	29.5	19.3	80.7	5.7	23.8	0.490	2.054
3% Ru/MXene-500 °C	43.5	12.4	87.6	5.4	38.1	0.437	3.072
3% Ru/MXene-550 °C	54.2	5.9	94.1	3.2	51.0	0.241	3.864
5% Ru/MXene-300 °C	0.1	100.0	0.0	0.1	0.0	0.015	0.000
5% Ru/MXene-350 °C	6.7	94.8	5.2	6.3	0.4	0.631	0.035
5% Ru/MXene-400 °C	17.9	80.2	19.9	14.3	3.6	1.326	0.328
5% Ru/MXene-450 °C	33.0	71.8	28.2	23.7	9.3	2.044	0.803
5% Ru/MXene-500 °C	42.6	57.4	42.6	24.4	18.2	1.971	1.465
5% Ru/MXene-550 °C	48.1	36.6	63.4	17.6	30.5	1.333	2.311

While other sheet-like materials such as graphene, rGO, or CNTs have been investigated as supports for Ru-based catalysts,^53,54^ MXenes offer a unique combination of features: high electrical conductivity, tunable surface functionalities, and hydrophilic surfaces that enhance metal dispersion and accessibility. Moreover, their structural and chemical versatility enables controlled metal–support interactions, reducing the likelihood of encapsulation while maintaining sufficient stabilization. These characteristics collectively contribute to the better performance of Ru/MXene observed in this study. The mechanistic role of MXene in the catalytic system can be primarily attributed to its two-dimensional structure, high electrical conductivity, and tunable surface chemistry, which together facilitate effective electron transfer and stabilize highly dispersed metal nanoparticles. The surface terminations (e.g., –O, –OH, and –F) can also modulate metal–support interactions, potentially enhancing catalytic activity and selectivity. In parallel, the incorporation of microalgae-derived residues introduces a sustainable and carbon-rich component, which can act as a physical support. The synergy between MXene and biomass-derived components provides a multifunctional platform that supports efficient catalytic performance, while contributing to greener synthesis strategies.

Furthermore, the STY for CH_4_ (Figure 4F), which measures the productivity of the catalyst in terms of CH_4_ production per mass of catalyst per hour, was found to be significantly higher for the 5% Ru/MXene catalyst, achieving a maximum for both catalysts at 450 °C. Specifically, the STY for the 5% Ru/MXene catalyst was 2.04 μmol CH_4_·g_cat_^–1^·h^–1^ compared to 0.49 μmol CH_4_·g_cat_^–1^·h^–1^ for the 3% Ru/MXene catalyst. Accordingly, at 550 °C, STY for CO was highest for 3% Ru/MXene at 3.86 μmol of CO·g_cat_^–1^·h^–1^ while for 5% Ru/MXene it reached 2.31 μmol of CO·g_cat_^–1^·h^–1^. Due to the nonlinear relationship between ln­(*k*) and 1/*T* across the studied temperature range, no apparent activation energy was extracted (Supporting Information Note 6, Figure S6). Instead, the rate–temperature dependence was fitted using an empirical Boltzmann function, which provided an improved correlation with the observed behavior.

These differences underscore the superior performance of the 5% Ru/MXene catalyst for CH_4_ production, likely due to enhanced active site availability, which can be utilized as a fuel or by methanotrophic microorganisms for biological processes, contributing to life support systems by recycling carbon and generating additional resources.^5^ Nevertheless, the produced CO also has the potential to be utilized as a valuable feedstock for synthesis processes, including the production of fuels and chemicals, which is particularly advantageous in space conditions where resources are limited and in situ space resource utilization is essential. The difference in selectivity on non-atomically dispersed Ru-based catalysts at similar CO_2_ conversions was described before.^14^ It was initially proposed that the catalysts selective to CO consist of only single-atom sites while those selective to CH_4_ contain mainly NPs; however, it was observed that charge transfer under reaction conditions plays a vital role, leading to changes in adsorption and intermediate activation, which subsequently determines the distinctly different selectivity.^14^

Chemisorption measurements were conducted to determine the dispersion of ruthenium, metal surface area (MSA), and surface metal atom density across different catalyst formulations (Supporting Information Note 7). The results are summarized for 5% Ru/MXene, 3% Ru/MXene, 6% Ru/TiO_2_, and 3% Ru/TiO_2_ catalysts (Table 3). The metal surface atoms per gram of catalyst and metal dispersion values revealed a clear dependence on both the Ru loading and support type. The amount of Ru surface atoms per gram of catalyst for 5% Ru/MXene was calculated to be 3.66 × 10^–7^ moles, while for 3% Ru/MXene it was 1.61 × 10^–7^. A lower value of 1.43 × 10^–7^ moles of Ru surface atoms was obtained for 6% Ru/TiO_2_ with 6.25 × 10^–8^ for 3% Ru/TiO_2_. Overall the Ru dispersion was quite low. The 5% Ru/MXene sample exhibited the highest value (1.34%), followed by 3% Ru/MXene (0.95%). In contrast, the TiO_2_-supported catalysts showed notably lower dispersions: 0.42% for 6% Ru/TiO_2_ and 0.38% for 3% Ru/TiO_2_. Correspondingly, the metal surface areas (MSA) followed the same trend. 5% Ru/MXene had the largest MSA (1.34 × 10^–2^ m^2^/g), whereas 3% Ru/MXene, 6% Ru/TiO_2_, and 3% Ru/TiO_2_ showed progressively lower values: 5.90 × 10^–3^ m^2^/g, 5.24 × 10^–3^ m^2^/g, and 2.29 × 10^–3^ m^2^/g, respectively. This decline with increasing Ru loading and switch to a TiO_2_ support is consistent with lower Ru accessibility. The surface metal atom density, defined as the number of exposed Ru atoms per square meter of Ru surface (atom/m^2^), also exhibited systematic variation. The highest value was observed for 5% Ru/MXene (1.21 × 10^16^ atom/m^2^), followed by 3% Ru/MXene (4.50 × 10^15^ atom/m^2^), 6% Ru/TiO_2_ (1.25 × 10^15^ atom/m^2^), and 3% Ru/TiO_2_ (8.51 × 10^14^ atom/m^2^). The values representing the density of accessible Ru atoms on the measured metal surface suggest a more densely populated surface for the MXene-supported catalysts. The differences in the surface metal atom density reflect Ru dispersion and the extent of metal accessibility.

**Table 3:** H_2_ Chemisorption-Derived Surface Characteristics of Ru-Based Catalysts after Reductive Pre-treatment

catalyst	volume of adsorbed gas at STP (cm^3^/g)	Mol of H_2_ adsorbed per gram of catalyst (mol/g)	Mol of surface atoms per gram of catalyst (mol/g)	metal dispersion (%)	metal surface area (m^2^/g)	surface metal atom density (atom/m^2^)
3% Ru/MXene	1.80 × 10^–3^	8.0 × 10^–8^	1.61 × 10^–7^	0.95	5.90 × 10^–3^	4.50 × 10^15^
5% Ru/MXene	4.10 × 10^–3^	1.8 × 10^–7^	3.66 × 10^–7^	1.34	1.34 × 10^–2^	1.21 × 10^16^
3% Ru/TiO_2_	7.00 × 10^–4^	3.1 × 10^–8^	6.25 × 10^–8^	0.38	2.29 × 10^–3^	8.51 × 10^14^
6% Ru/TiO_2_	1.60 × 10^–3^	7.1 × 10^–8^	1.43 × 10^–7^	0.42	5.24 × 10^–3^	1.25 × 10^15^

In light of the TEM-derived average Ru particle sizes around 9 nm, the low dispersion suggests their (partial) encapsulation by MXene, while still maintaining exposure of active Ru atoms. In contrast, stronger metal–support interactions and higher porosity of TiO_2_ lead to deeper incorporation and total encapsulation, reducing Ru surface accessibility. Overall, these findings demonstrate that both Ru loading and support type significantly influence the dispersion, surface accessibility, and spatial distribution of the active Ru sites. The better dispersion and higher Ru atom density on MXene supports thus indicate better utilization of Ru and enhance catalytic performance in CO_2_ hydrogenation.

The turnover frequency (TOF) values were calculated based on the apparent reaction rates and the number of accessible surface Ru atoms obtained from H_2_ chemisorption (Supporting Information Note 7, Table S5). The 3% Ru/MXene sample exhibited higher TOF values overall, varying from 0.21 to 1.39 s^–1^. In contrast, for the 5% Ru/MXene catalyst, the TOF values ranged from 0.02 to 0.54 s^–1^ as the reaction temperature increased, indicating a moderate rise in intrinsic activity with temperature. This suggests that, despite its lower metal loading, 3% Ru/MXene has a higher proportion of accessible active sites with better intrinsic activity, likely due to improved dispersion and reduced metal aggregation. These values compare favorably with literature-reported TOFs for Ru-based catalysts, which typically range from 0.04 to 5.7 s^–1^ depending on the support material and reaction conditions.^55^ Commonly used P25 TiO_2_ contains a mixture of anatase and rutile phases, significantly influencing the catalytic performance.^56^ The presence of rutile has been associated with influencing interfacial compatibility with ruthenium, which may contribute to higher intrinsic activity.^22^

In our study, the MXene support undergoes controlled oxidation, forming TiO_2_ domains. This transformation is significant, as anatase TiO_2_ has been shown to affect the activity of supported Ru catalysts in CO_2_ hydrogenation reactions. Due to more favorable metal–support interactions and active site exposure, Ru nanoparticles supported on anatase TiO_2_ with predominant (101) facets have demonstrated higher TOFs than those on (001) facets, highlighting the potential contribution of facet-specific effects.^57^ The presence of anatase TiO_2_ (101) on the oxidized MXene surface may, therefore, enhance both the dispersion and intrinsic activity of Ru active sites. The anatase TiO_2_ domains formed on MXene may play a pivotal role in the observed catalytic performance, potentially through favorable facet exposure and unique metal–support interactions.

Building on the insights gained from kinetic measurements, *operando* DRIFTS of CO_2_ hydrogenation was used as an analytical technique to characterize adsorbed species present during reaction (Figure 4G), in an effort to understand the active sites involved and to obtain hints on the reaction mechanism (Figure 5). *Operando* DRIFTS spectra of MXene-supported catalysts at 400 °C revealed peaks at 3017 cm^–1^ and 1303 cm^–1^ originating from C–H bonds of CH_4_, adsorbed formate species (*HCO_2_) that appeared at 1560 cm^–1^, while a formyl peak *CHO was observed at 1760 cm^–1^, as well as adsorbed carbonate (*HCO_3_) at 1360 cm^–1^.^22,58^ As the temperature increased to 400 °C, new bands appeared at 1930 cm^–1^, corresponding to bridged carbonyl species Ru–(CO)_2_.^59^ Notably, a significant increase in the intensity of methoxy species (*CH_3_O, 2950 cm^–1^)^60,61^ was observed at temperatures above 400 °C, indicating the formation of *CH_3_O as another key intermediate in the methanation pathway.^61–63^ The 5% Ru/MXene demonstrated a stronger and broader appearance of the carbonate species *HCO_3_, formed when adsorbed CO_2_ reacts with surface hydroxyl groups or hydrogen on the support as well as a more intense bridged carbonyl species Ru–(CO)_2_ band as the temperature increased.^58,64^ The formation of the methoxy species was significantly more pronounced at higher temperatures, indicating that the higher Ru loading enhances the stabilization of methoxy intermediates. Although *CH_3_O species are detected at temperatures above 400 °C, their presence does not directly correspond to CH_4_ formation, which is more selective at lower temperatures. This suggests that methoxy species may represent nonreactive intermediates under these conditions or accumulate due to altered surface kinetics. Therefore, their role in the methane production pathway requires careful interpretation and may not reflect a direct mechanistic link.

**Figure 5:**
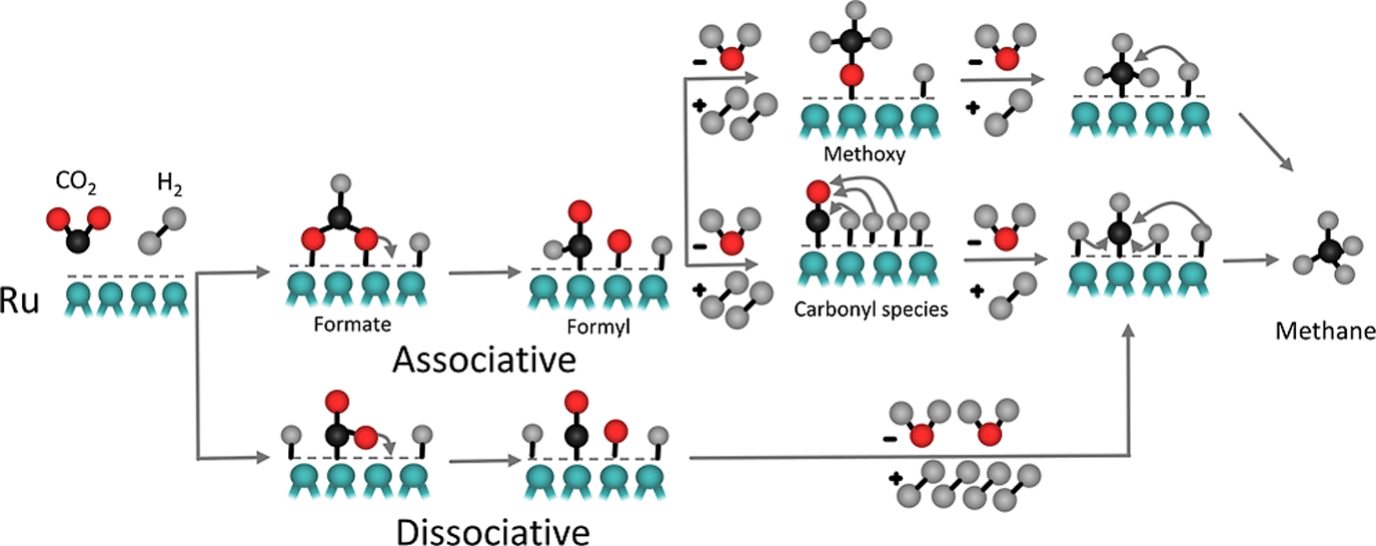
Proposed schematic mechanism of CO_2_ hydrogenation to methane on Ru-based catalysts. The support is omitted to simplify the graphics.

### 3.8 Reaction Pathway and Mechanistic Overview

Two mechanisms have been proposed for CO_2_ methanation. One suggests that CO_2_ is associatively adsorbed with H, while the other describes that CO_2_ first dissociates to CO and O (Figure 5). The typical CO_2_ hydrogenation pathway for Ru-based catalysts can follow both associative and dissociative CO_2_ methanation mechanisms involving the sequential adsorption and hydrogenation of CO_2_ on the catalyst surface to finally produce methane.^62^ Initially, CO_2_ molecules adsorb onto active sites of the catalyst, such as under-coordinated metal atoms, and the adsorbed CO_2_ undergoes a series of hydrogenation steps. First, CO_2_ is hydrogenated to form the *HCO_2_ formate intermediate, which further reacts to produce formyl *CHO, followed by the formation of *CH_3_O intermediates.^62^ The overall process involves the breaking of C=O bonds and the formation of C–H bonds, facilitated by the transfer of hydrogen atoms from the catalyst surface.^62^ The presence of specific catalytic sites and the effective stabilization of reaction intermediates are crucial for the efficient conversion of CO_2_ to CH_4_ via the associative and dissociative pathway.

The associative and dissociative mechanisms in CO_2_ hydrogenation on Ru can be interconnected through reaction intermediates, particularly CO. In the associative pathway, CO_2_ hydrogenates stepwise to formate or methoxy intermediates, which can further hydrogenate to methane. However, on Ru, these species can also decompose to CO, linking the associative pathway to the dissociative mechanism, where CO undergoes further hydrogenation to CH_4_. The interconnection shows that while some CO_2_ may directly hydrogenate to methane via associative steps, a significant portion dissociates into CO and O, with CO following a separate hydrogenation route. The balance between these pathways depends on the reaction conditions, catalyst properties, and metal–support interactions. It was also found that Ru/RuTiO_2_ favors the associative pathway, where CHO* hydrogenates further to CH_2_O* due to its greater stability and a lowered d-band center, while Ru/TiO_2_ follows a dissociative pathway, preferring direct C–O bond cleavage after isomerization, driven by the thermodynamic stability of [CH + O]* and an uplifted d-band center.^65^

The observed trend that lower Ru loading leads to more CO production, while higher loading favors methane formation, can be explained by the availability of active sites and reaction pathways. At lower loading, the active sites may favor the formation of CO through the initial activation of CO_2_, leading to dicarbonyl formation. However, at higher Ru loading, the availability of active sites facilitates further hydrogenation of CO, thus favoring methane formation over the production of heavier hydrocarbons. The change allows for a more efficient conversion of CO_2_ to methane as the reaction progresses, highlighting the importance of optimizing catalyst loading to balance the production of CO and CH_4_ through the intermediates. The findings are consistent with the literature, describing the hydrogenation of carbonyl to methane as a rate-limiting step of the pathway.^62^ The CO_2_ hydrogenation pathways over the Ru(0001) surface were also investigated by computational modeling.^66,67^ The results showed that direct CO_2_ dissociation was only thermodynamically favorable but needed to overcome a high energy barrier to occur (*E*_a_ = 1.20 eV), whereas the pathway for C–O bond breaking by the *HCO_2_ route (*E*_a_ = 0.37 eV) was feasible, which further dissociated to the *CHO intermediate (*E*_a_ = 0.41 eV).^66^ While the study provides a valuable understanding of the progression of intermediates, clear confirmation of the role of the observed surface species as active intermediates, rather than being spectators, would require advanced techniques such as isotopic labeling studies using steady-state isotopic transient kinetic analysis (SSITKA)-DRIFTS-MS^68^ or modulation excitation spectroscopy.^15,59,69–72^ The provided insights may help in the design of more efficient catalysts and optimization of reaction conditions for improved CO_2_ hydrogenation performance.

### 3.9 Encapsulation Behavior and SMSI Effects

Finally, the low/absent activity of Ru NPs supported on the TiO_2_ anatase, which was reported before, needs to be explained.^22^ RuO_2_ NPs are observed to be well-dispersed within the porous TiO_2_ support, which may limit the accessibility of some active sites due to physical confinement. However, under reducing conditions typical of reaction environments, partial strong metal–support interaction (SMSI) effects may arise, especially in defect-rich or nanostructured anatase. While the initial adhesion between Ru and the anatase surface seems weak, thermally induced changes during reaction may lead to dynamic restructuring and electronic interactions characteristic of SMSI. Combined with the high reducibility of surface oxygen atoms, the likelihood of a SMSI effect (encapsulation) is enhanced, hindering CO adsorption and influencing catalytic activity and product selectivity (from CH_4_ to CO). In contrast, the MXene structure provides multiple interfaces, including TiC and TiO_2_, that can influence metal–support interactions (MSI), promoting better electron transfer between the metal and the support as well as between the catalyst and reactants, leading to enhanced activation of reactants. Additionally, the MXene surface atomic configurations can create more reactive sites than pure TiO_2_ anatase, leading to a higher catalytic performance. The MSI and the distinctive surface geometry increase the availability of active Ru sites, facilitating more efficient CO_2_ hydrogenation and CH_4_ production.^73^ Furthermore, the TiC component in MXene-TiO_2_ seems to reduce the extent of encapsulation (or hinder it), creating a synergy between accessible metal sites and beneficial support interactions. Thus, TiC may help to maintain or even enhance activity.

Thus, 6% RuO_2_/TiO_2_ was also tested for CO_2_ hydrogenation after pretreatment in O_2_ at 300 °C followed by reduction in H_2_ at 300 °C (Table S4). However, CO_2_ conversion ranges between 1 and 4%, slightly increasing upon rising temperatures. It is possible that while the catalyst has been reduced, the nature of the active sites formed may not be sufficient due to weak metal–support interactions or a lack of suitable active sites for CO_2_ activation. The results confirm the previous studies highlighting the role of Ru encapsulation by TiO_*x*_ layers and hydroxyl groups on TiO_2_ for CO_2_ activation, which require higher reduction temperatures than indicated by H_2_-TPR.^49^

TEM analysis provided critical insights into the potential encapsulation of Ru NPs on MXene and TiO_2_ supports after H_2_ reduction at 550 °C of 5% RuO_2_/MXene and 6% RuO_2_/TiO_2_ (Figure 6). TEM analysis after H_2_ reduction revealed a significant decrease in particle size, suggesting restructuring and possible redispersion of the metallic phase. On MXene, the average Ru particle size decreased to 9.21 nm, with improved dispersion and fewer aggregates compared to the oxidized state. The layered morphology and surface functional groups of MXene likely facilitated redistribution and stabilization of smaller Ru nanoparticles during reduction. On TiO_2_, the particle size also decreased, albeit to a lesser extent, with an average diameter of 17.7 nm. Size distribution histograms before and after reduction are provided in Figures S4 and S7. Additionally, the interactions between Ru nanoparticles and the MXene may limit the encapsulation process by influencing Ti species mobility, altering local chemical environments, and introducing physical barriers due to MXene’s layered structure. While both supports provide direct contact between Ru and TiO_2_, MXene disrupts the uniform migration of TiO_*x*_ species during reduction, resulting in only *partial* encapsulation that leaves more Ru sites accessible for catalysis compared to the complete encapsulation observed on pure TiO_2_.

**Figure 6:**
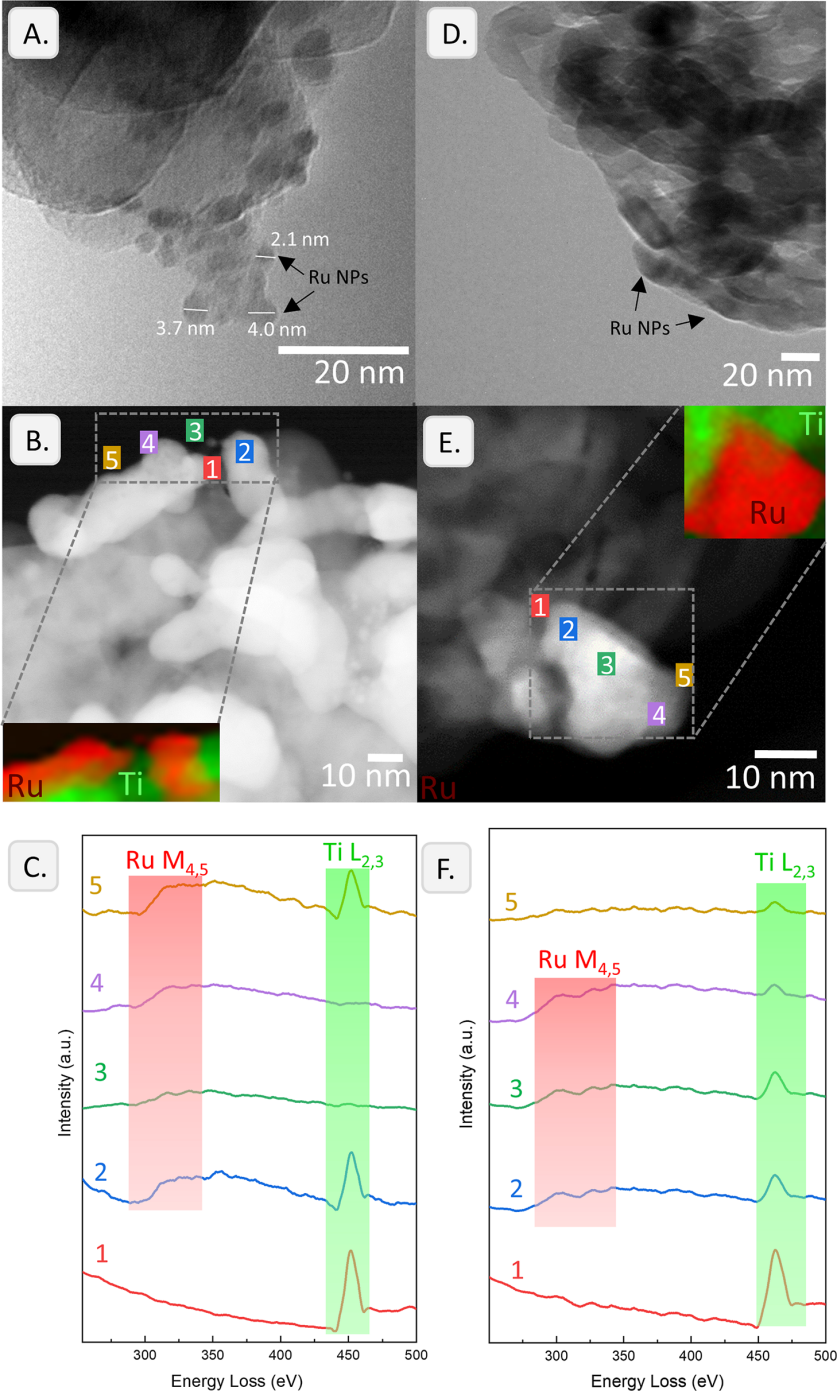
Surface morphology and encapsulation analysis of Ru NPs on different supports. (A) HR-TEM image and (B) STEM image with overlaid EELS elemental mapping of 5% Ru/MXene, (C) EELS spectra from five selected areas of 5% Ru/MXene, (D) HR-TEM images and (E) STEM image with overlaid EELS elemental mapping of 6% Ru/TiO_2_, and (F) EELS spectra from five selected areas of 6% Ru/TiO_2_.

On the TiO_2_ support, the Ru nanoparticles were consistently encapsulated by a continuous TiO_*x*_ layer, with a uniform thickness across all observed particles. The observed uniformity suggests that Ti species from the TiO_2_ support migrated effectively to the Ru surface during the reduction process, forming a stable protective layer. In contrast, many Ru nanoparticles on the MXene support showed partial coverage by TiO_*x*_, with some particles remaining exposed or displaying thinner, nonuniform layers. The variation in encapsulation likely stems from the layered structure and chemical nature of MXene, which may disrupt the migration of the Ti species and alter the growth dynamics of the TiO_*x*_ layer.

These structural differences align with the observed catalytic performance, where Ru on MXene demonstrated superior activity compared with the fully encapsulated Ru on TiO_2_. The partial encapsulation on MXene allows for greater accessibility of Ru active sites, likely contributing to its enhanced performance. In comparison, the complete encapsulation of Ru on TiO_2_ inhibits the catalytic activity by blocking surface sites required for the reaction. Thus, the extent of encapsulation, as revealed by TEM, plays a significant role in determining the catalytic behavior of the Ru nanoparticles.

## 4 Conclusions

This study demonstrated how Ru can be loaded on MXene or TiO_2_ supports by using microalgal extracts, yielding catalysts for the CO_2_ hydrogenation. While the MXene support showed a lower specific surface area and less homogeneous RuO_2_ NPs dispersion (in the precatalyst) than TiO_2_, after reductive pretreatment, it exhibited superior catalytic activity, indicating that the unique textural properties and active site availability compensates for the lower surface area. Microscopy studies indicated different morphologies, with MXene showing accordion-like structures and TiO_2_ exhibiting spherical particles, influencing the dispersion and interaction of RuO_2_ NPs on the respective supports. The specific reduction behavior observed in H_2_-TPR revealed that MXene-supported catalysts exhibit multiple reduction peaks, indicating a reduction involving diverse catalytic sites and different surface species, more complex than the single reduction peak on the TiO_2_ support. This is confirmed by the various and more intense CO–DRIFTS peaks associated with binding of the CO molecules to Ru on MXene, suggesting bridge-binding catalytic sites, possibly contributing to enhanced CO_2_ hydrogenation activity. In contrast, TiO_2_ displays a twin CO binding environment, demonstrating the advantages of utilizing MXene as a support material for improved catalytic performance. A higher Ru loading on MXene resulted in higher methane selectivity and conversion than lower loading, highlighting the importance of optimizing catalyst loading for enhanced performance. Lower Ru loading on MXene showed a preference for CO formation. Beyond catalytic performance, this work provides mechanistic insights into the role of bioresidues in enhancing metal dispersion and highlights the surface and electronic contributions of the MXene support. The environmentally benign synthesis route, combined with detailed structural and spectroscopic characterization, offers a sustainable and tunable platform for designing future catalysts. Clear confirmation of the observed surface species as active intermediates, rather than spectators, requires advanced techniques such as isotopic labeling studies or specialized spectroscopic methods. Overall, the results underscore the promise of using MXene as a support material for metal catalysts in CO_2_ hydrogenation, paving the way for future research to explore the stability and recyclability of these catalysts in practical applications.

## Supplementary Material



## Notes

Additional calculations from the study, details of the methodology, chemical composition, TEM image analysis, XPS spectra, and catalytic activity (PDF)
