# Supplementary material for: Influence of MXene and TiO2 on the Performance of Microalgae-Derived Ru-Based Catalysts for CO2 Hydrogenation to Methane
Source: ACS Catal. 2025 Aug 19;15(17):15261–78. doi: 10.1021/acscatal.5c04285 (PMC12418307; doi:10.1021/acscatal.5c04285)
Supplement: Supplementary file 1 [file cs5c04285_si_001.pdf]

Influence of MXene and TiO_2 on the Performance of Microalgae-Derived Ru-Based Catalysts for CO_2 Hydrogenation to Methane

Agnieszka Sidorowicz[1,2], Thomas Wicht[2], Michael Stöger-Pollach[3], Roberta Licheri[1,4], Giacomo Cao[1,4,5], Alessandro Concas[1,4,], Günther Rupprechter[2,]**

[1]*Interdepartmental Centre of Environmental Engineering and Sciences, University of Cagliari, Cagliari, 09123 Cagliari, Italy*

[2]*Institute of Materials Chemistry, TU Wien, Getreidemarkt 9/BC, Vienna, 1060 Vienna, Austria*

[3]*University Service Center for Transmission Electron Microscopy, TU Wien, Stadionallee 2/057-02, Vienna, 1020 Vienna, Austria*

[4]*Department of Mechanical, Chemical and Materials Engineering, University of Cagliari, Piazza d'Armi, Cagliari, 09123 Cagliari, Italy*

[5]*Center for Advanced Studies, Research and Development in Sardinia (CRS4), Loc. Piscina Manna, Building 1, Pula, 09050 Pula, Italy.*

**Corresponding author(s)*

alessandro.concas@unica.it (A. Concas)

guenther.rupprechter@tuwien.ac.at (G. Rupprechter)

Supplementary Note 1:

The nominal loadings were calculated based on the following equations:

$m_{Ru} = \frac{m_{RuCl_3} \times \mathrm{M_{Ru}}}{M_{RuCl_3}}$ ……. (Eq. 1)

where:

m_{Ru} = Ru mass (g)

m_{RuCl_3} = $RuCl_3$ mass (g)

M_{Ru} = Ru molar mass (g/mol)

M_{RuCl_3} = $RuCl_3$ molar mass (g/mol)

Then, the obtained values were converted to moles:

$n_{Ru} = \frac{m_{Ru}}{M_{Ru}}$ ……. (Eq. 2)

where:

n_{Ru} = Ru moles (mol)

Followed by oxygen mass calculations:

$m_O = n_{Ru} \times 2 \times M_O$……. (Eq. 3)

where:

m_O = O mass (g)

M_O = O molar mass (g/mol)

The mass of the RuO2 component was then obtained:

$m_{RuO_2} = m_{Ru} + m_O$ ……. (Eq. 4)

where:

m_{RuO_2} = RuO_2 mass (g)

Finally, the nominal loading was calculated based on:

$\%_{Ru_N} = \frac{m_{Ru}}{m_{RuO_2}+1.5} \times 100$ ……. (Eq. 5)

where:

$\%_{Ru_N}$= nominal Ru percentage (%)

Supplementary Note 2:

The calibrated peak areas from GC chromatograms were utilized to calculate CO_2 conversion (Eq.6):

$$Conversion_{CO_2}(\%) = \frac{A^{in}_{CO_2} - A^{out}_{CO_2}}{A^{in}_{CO_2}} \times 100 \text{ .... (Eq.6)}$$

with:

$A^{in}_{CO_2}$= Peak Area of CO_2 entering the reactor

$A^{out}_{CO_2}$ = Peak Area of CO_2 exiting the reactor

The selectivity of the catalysts for producing CH_4 $Selectivity_{CH_4}(\%)$ was determined using Eq.7:

$$Selectivity_{CH_4}(\%) = \frac{A_{CH_4}}{A_{CH_4} + A_{CO}} \times 100 \text{ .... (Eq.7)}$$

where:

A_{CH_4} = Peak area of CH_4

A_{CO_2} = Peak area of CO_2

While CO selectivity was determined using Eq. 8:

$$Selectivity_{CO}(\%) = \frac{A_{CO}}{A_{CH_4} + A_{CO}} \times 100 \text{ .... (Eq.8)}$$

With conversion (Eq. 6) and selectivity (Eq. 7 or 8), the yield of CH_4 or CO can be calculated using Eq. 9, and Eq. 10:

$$Yield_{CH_4}(\%) = \frac{Conversion_{CO_2} \times Selectivity_{CH_4}}{100} \text{ .... (Eq. 9)}$$

$$Yield_{CO}(\%) = \frac{Conversion_{CO_2} \times Selectivity_{CO}}{100} \text{ .... (Eq. 10)}$$

To measure the interaction between the reactants H_2/CO_2 and the catalyst bed in the reactor, it is necessary to determine the gas hourly space velocity (GHSV) describing the amount of CO_2 that passes through the catalyst bed per hour. Therefore, the total volumetric flow of the reactants, as well as the volume of the catalyst bed, are used for its calculation, as shown in Eq. 11:

$$GHSV = \frac{\dot{V}_{Reactant}}{V_{Catalyst}} = \frac{\dot{V}_{CO_2}}{\pi \times r^2 \times h} \text{ .... } (Eq.\ 11)$$

where:
GHSV = Gas Hourly Space Velocity (h^{-1})
$\dot{V}_{Reactants}$ = Volumetric flow rate of reactant (0.75 L/h)
$V_{Catalyst}$ = Volume of catalytic bed (1.5×10^{-4} L)
r = internal radius of reactor (4×10^{-2} dm)
h = height of catalytic bed (3×10^{-2} dm)
The calculated GHSV had a value of 5000 h^{-1}. Moreover, the reciprocal value of the GHSV is the residence time (τ) based on Eq. 12:

$$\tau = \frac{1}{GHSV} \times 3600 \text{ .... (Eq. 12)}$$

In this case, a reactant molecule spends 0.72 seconds in the catalyst bed before leaving the reactor.

The space-time yield (STY) was used to characterize the performance of the catalysts. To determine this, the volume of the reagent gas is calculated according to Eq. 13 using the reaction time and the gas flow rate:

$\mathrm{V}_{CO_2} = \dot{\mathrm{V}}_1 \times \mathrm{t}$ .... (Eq. 13)

with:

v_{CO_2} = volume of CO_2 (L)

V_1 = total gas flow of CO_2 (2.5 mL/min = 0.15 L/h)

t= residence time (0.0002 h)

The molar amount of the product, which equals that of the reagent gas, is determined by rearranging the universal gas equation (Eq. 14):

$n_{CO_2} = \frac{p \times \mathrm{V}_{CO_2}}{\mathrm{R} \times T} = n_p$ .... (Eq. 14)

where:

n_{CO_2} = amount of CO_2 (mol)

p = reaction pressure (1 bar)

v_{CO_2} = volume of CO_2 (L)

R = universal gas constant (0.083143 L×bar×mol^{-1}×K^{-1})

T = reaction temperature (K)

n_p = amount of product (mol)

The STY values are finally obtained by inserting the theoretical CH_4 or CO yield into Eq. 15:

$STY = \frac{Yield \times n_p}{100 \times m_{cat} \times \mathrm{t}}$....... (Eq. 15)

with:

STY= space-time yield ($\mu mol_{product} \times g_{cat}^{-1} \times h^{-1}$)

Yield = measured yield (%)

n_p = amount of product (mol)

m_{cat} = mass of catalyst (0.02 g)

t = residence time (0.0008 h)

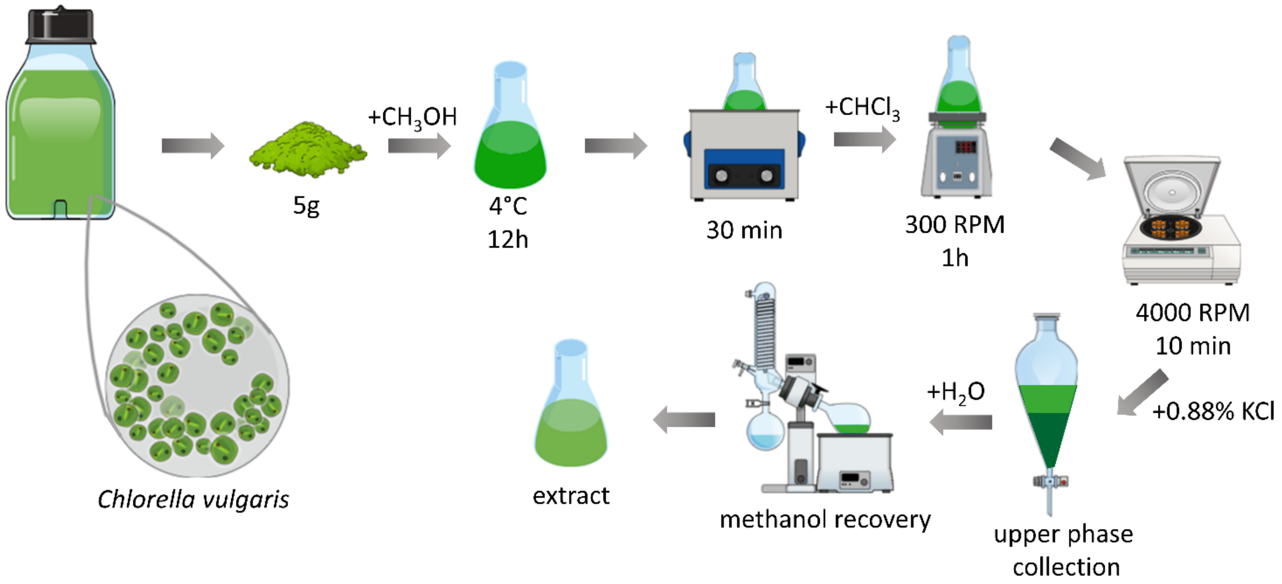

Figure S1 Extract preparation.

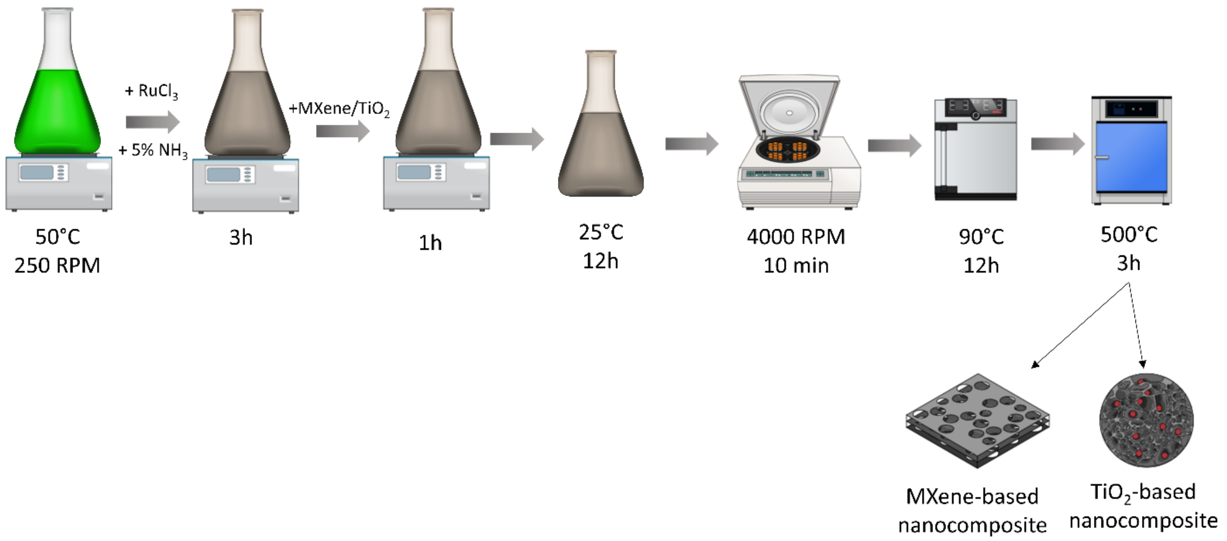

Figure S2 Impregnation method.

Table S1 Composition of the obtained catalysts based on XRF results (wt.%)

Catalyst	Composition (%)					
	Ru	Ti	O	Al	Na	F
MXene	0	74.6	21.2	1.4	0.6	2.2
3% RuO_2/MXene	3.3	73.4	22.1	1.1	0.2	1.0
5% RuO_2/MXene	5.2	68.7	23.6	1.1	0.2	1.1
TiO_2	0	69.7	30.3	0	0	0
3% RuO_2/TiO_2	3.4	70.7	25.9	0	0	0
6% RuO_2/TiO_2	6.5	66.0	27.6	0	0	0

Supplementary Note 3:

The actual Ru loading was calculated based on the following equations:

$m_{Ru} = \%_{Ru_{XRF}} \times m_{RuO_2} + 1.5$ ……. (Eq. 16)

Where:

$\%_{Ru_{XRF}}$ = Ru percentage from XRF analysis (%)

The next calculations followed a similar methodology as nominal loading:

$n_{Ru} = \frac{m_{Ru}}{M_{Ru}}$ ……. (Eq. 17)

$m_O = n_{Ru} \times 2 \times M_O$ ……. (Eq. 18)

$m_{RuO_2} = m_{Ru} + m_O$……. (Eq. 19)

$\%_{Ru_A} = \frac{m_{Ru}}{m_{RuO_2}+1.5} \times 100$……. (Eq. 20)

where:

$\%_{Ru_A}$= actual Ru percentage (%)

Table S2 Calculated nominal and actual loadings.

Catalyst	Nominal Ru loading (%)	Nominal RuO_2 loading (%)	Actual Ru loading (%)	Actual RuO_2 loading (%)
3% RuO_2/MXene	3.1	4.0	3.3	3.2
5% RuO_2/MXene	5.9	7.8	5.2	4.8
3% RuO_2/TiO_2	3.1	4.0	3.4	3.3
6% RuO_2/TiO_2	5.9	7.8	6.5	6.1

Table S3 Composition of the obtained catalysts based on XPS results (at.%)

Catalyst	Composition (%)					
	Ru 3d	Ti	O	Al	Na	F
3% RuO_2/MXene	2.2	27.0	62.9	3.7	0.2	4.3
5% RuO_2/MXene	3.5	25.6	63.0	3.6	0.2	4.2
3% RuO_2/TiO_2	1.4	29.8	68.4	0	0	0
6% RuO_2/TiO_2	1.9	28.7	68.8	0	0	0

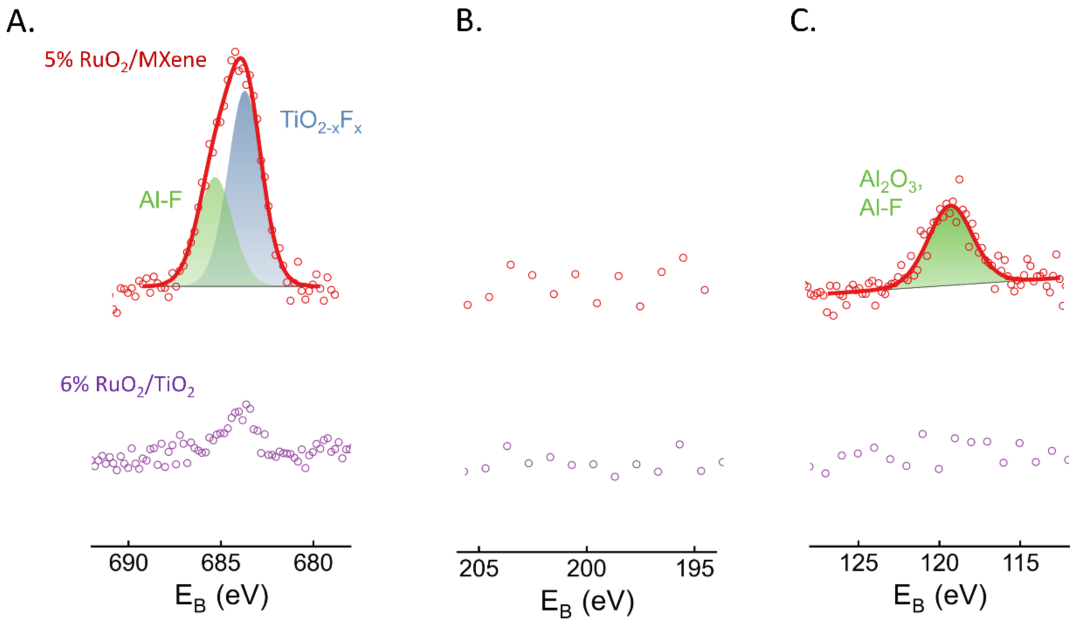

Figure S3 XPS spectra of 5% RuO_2/MXene and 6% RuO_2/TiO_2: (A) F 1s, (B) Cl 2p, and (C) Al 2s regions.

Supplementary Note 4:

The surface area of an average spherical particle with a 14 nm diameter was calculated based on the following equation:

$A = 4\pi r^2$……(Eq. 21)

r = sphere radius (7 nm)

A = sphere surface area (615.4 nm^2)

The volume of the average particle was obtained using:

$V = \frac{4}{3}\pi r^3$……(Eq. 22)

V= 1436.0 nm^3 = 1.436e-18 cm^3

ρ = RuO_2 density (6.97 g/cm^3)

The mass of the average particle was calculated by:

$m = \rho \times V$……(Eq. 23)

m = mass of the average RuO_2 particle (1.0e-17 g)

In the next step, the weight of RuO_2 per gram of the catalyst was obtained:

$m_{RuO_2} = \frac{\%_{Ni} \times M_{RuO_2}}{M_{Ru}}$……(Eq. 24)

m_{RuO_2} = RuO_2 mass (g)

$\%_{RuO_2}$ = Ru percentage (6, 5, or 3%)

M_{RuO_2} = RuO_2 molar mass (133.07 g/mol)

M_{Ru} = Ru molar mass (101.7 g/mol)

Given the obtained total RuO_2 weight, the number of RuO_2 particles per gram of catalyst was calculated as:

$N_{RuO_2} = \frac{m_{RuO_2}}{m}$……(Eq. 25)

N_{RuO_2} = number of RuO_2 nanoparticles per gram catalyst (7.84E+15 for 6% Ru, 6.54E+15 for 5%Ru, and 3.92E+15 for 3%Ru)

Finally, the total surface area of the RuO_2 nanoparticles per gram catalyst was obtained:

$SS_{RuO_2} = N_{RuO_2} \times A$……(Eq. 26)

SS_{RuO_2} = RuO_2 surface area (nm^2) per gram catalyst (4.8E+04 nm^2 for 6%Ru, 4.0E+04 nm^2 for 5%Ru, and 2.4E+04 nm^2 for 3%Ru)

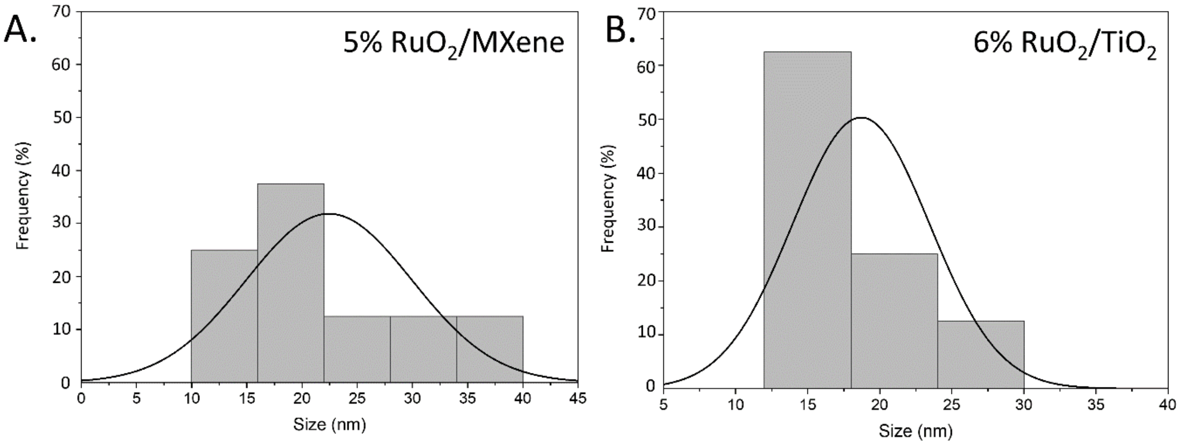

Figure S4 Size distribution histograms: (A) RuO_2/MXene, (B) RuO_2/TiO_2.

Table S4 Overview of CO_2 conversion, product selectivity (CH_4 and CO), and yield, for 6% RuO_2/TiO_2 at different temperatures.

Catalyst	CO_2 Conversion (%)	CH_4 Selectivity (%)	CO Selectivity (%)	CH_4 Yield (%)	CO Yield (%)
6% Ru/TiO_2-300°C	0.0	0	0	0	0
6% Ru/TiO_2-350°C	1.1	77.1	22.9	0.9	0.3
6% Ru/TiO_2-400°C	2.0	32.7	67.3	0.7	1.3
6% Ru/TiO_2-450°C	1.9	21.6	78.4	0.4	1.5
6% Ru/TiO_2-500°C	3.6	17.4	82.6	0.6	3.0
6% Ru/TiO_2-550°C	3.7	0.0	100.0	0.0	3.7

Supplementary Note 5:

To quantify the deactivation behavior, the decline in catalytic activity over time was modeled using a first-order exponential decay function (Eq. 27), as it is applied to describe catalyst deactivation processes dominated by a single-path mechanism, such as gradual loss of active sites:

$A(t) = y_0 + A_1 e^{-t/t_1}$......(Eq. 27)

where:

A(t) = the catalytic activity at time t, residual activity (%),

y_0 = the amplitude of decay (%),

t_1 = the decay time constant (h).

The fitting was performed using OriginPro software (Fig. S5). The quality of fit was confirmed by regression analysis (R^2 = 0.966). The fitted parameters (y_0=39.05%, A_1=61.91%, and t_1=59.74 h) provided a quantitative basis for estimation of the residual activity plateau, the characteristic timescale of catalyst deactivation, as well as extrapolating the long-term stability.

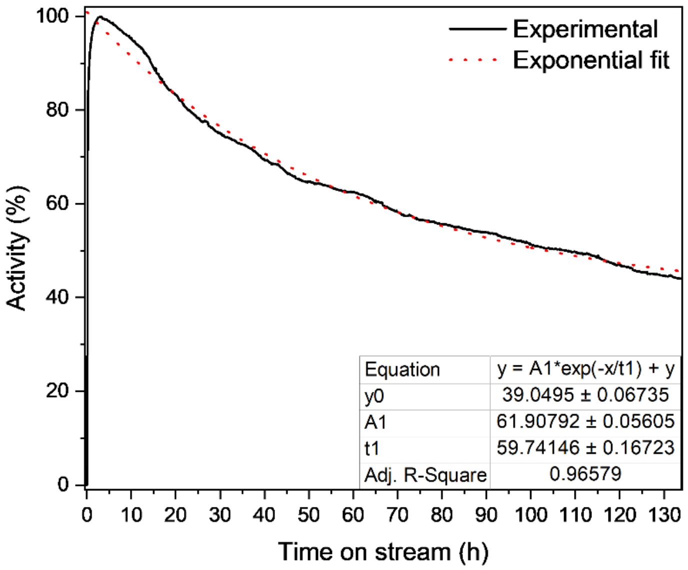

Figure S5 Catalyst deactivation over time fitted with an exponential decay model alongside experimental data.

Supplementary Note 6:

We have calculated the apparent reaction rate based on the equation below (Eq. 28):

$$r_{app} = \frac{GHSV \times Conversion_{CO_2} \times V_{cat}}{V_M * m_{cat}} \text{......(Eq. 28)}$$

where:

r_{app}= Apparent reaction rate (mol CO_2 converted per hour per gram)

GHSV = Gas Hourly Space Velocity (h^{-1})

$Conversion_{CO_2}$ = CO_2 conversion (fractional)

V_{cat} = Volume of the reactants (ml)

V_M = Molar volume at given temperatures (ml/mol)

m_{cat} = catalyst mass (g)

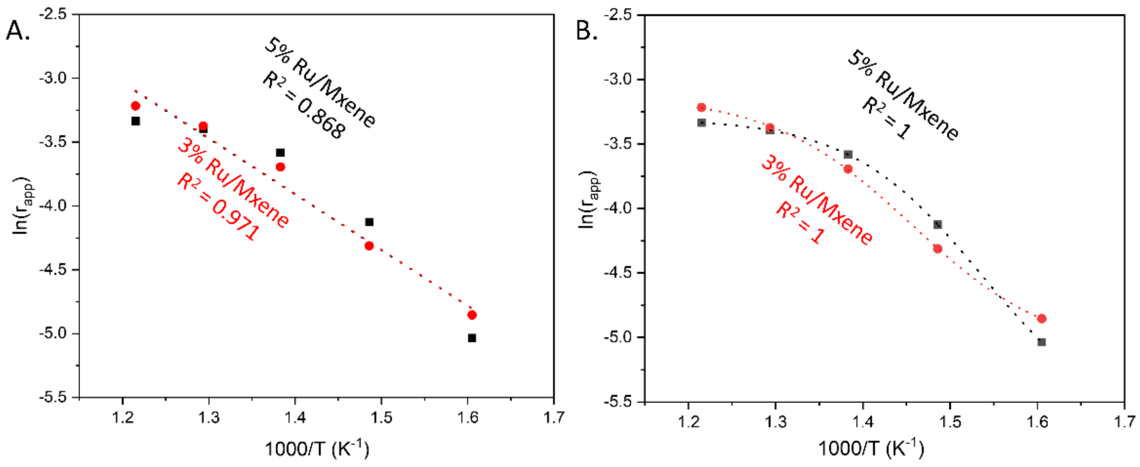

Figure S6 Arrhenius plots: (A) linear fit, (B) Boltzmann fit.

Supplementary Note 7:

The calculations below were performed based on the chemisorption data. First, moles of adsorbed H_2 per gram of catalyst were calculated based on Eq. 29:

$n_{H_2} = \frac{V_{ads}}{V_{mv}}$……(Eq. 29)

where:

n_{H_2} = moles of H_2 adsorbed per gram of catalyst (mol/g)

V_{ads} = volume of adsorbed gas at STP (cm^3/g)

V_{mv} = molar volume of gas at STP (22414 cm^3/mol)

Based on the obtained values, moles of surface Ru atoms per g of catalyst were calculated (Eq. 30):

$n_{surface} = n_{H_2} \times SF$……(Eq. 30)

where:

$n_{surface}$ = moles of surface atoms per g of catalyst (mol/g)

SF = stoichiometric factor (2 for H_2 adsorption on Ru)

Next, Ru dispersion was calculated using Eq. 31:

$D_m(\%) = \frac{n_{surface} \times M_{metal}}{w_{metal} \times m_{cat}} \times 100\%$……(Eq. 31)

where:

$D_m(\%)$ = metal dispersion (%)

M_{metal} = molar mass of the metal (101.07 g/mol for Ru)

w_{metal} = metal weight fraction/loading (fractional)

m_{cat} = catalyst mass (g)

Having established the metal dispersion, the metal surface area was subsequently calculated to determine the active surface available for catalytic interactions (Eq. 32):

$MSA = n_{surface} \times N_A \times A_{metal}$……(Eq. 32)

where:

MSA = metal surface area (m^2/g)

N_A = Avogadro's number (mol^{-1})

A_{metal} = atomic cross-sectional area of metal (0.061 nm^2 for Ru)

From the calculated metal surface area, the surface metal atom density was estimated to quantify the number of active metal atoms per unit area (Eq. 33):

$\rho_{surface} = \frac{n_{surface} \times N_A}{SS_{BET}}$……(Eq. 33)

where:

$\rho_{surface}$ = surface metal atom density (atom/m^2)

SS_{BET} = surface area from BET method (m^2/g)

Turnover frequency was subsequently determined by relating the reaction rate to the number of surface metal atoms, providing a measure of the catalytic efficiency on a per-site basis (Eq. 34):

$TOF = \frac{r_{app}}{n_{surface} \times m_{cat}} \div 3600$……(Eq. 34)

where:

TOF = turnover frequency (s^{-1})

Table S5 Catalytic performance of Ru/MXene catalysts at different temperatures. Ru/TiO_2 was inactive.

Catalyst	Temperature (°C)	Apparent Rate (mol CO_2/h×g)	Turnover frequency (s^{-1})
3% Ru/MXene	300	0.0061	0.2096
	350	0.0078	0.2698
	400	0.0134	0.4636
	450	0.0249	0.8597
	500	0.0343	1.1853
	550	0.0401	1.3868
5% Ru/MXene	300	0.0001	0.0022
	350	0.0065	0.0988
	400	0.0162	0.2454
	450	0.0278	0.4223
	500	0.0336	0.5096
	550	0.0356	0.5405

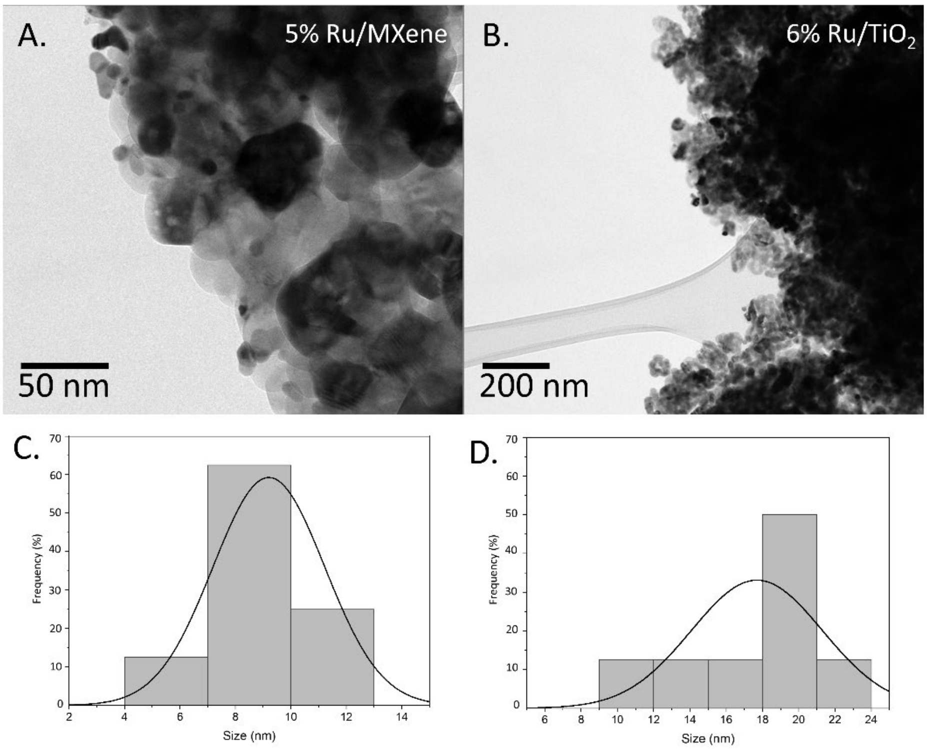

Figure S7 TEM micrographs and corresponding particle size distribution histograms of Ru nanoparticles on (A, C) MXene and (B, D) TiO_2 supports.
